# Short-pulsed micro-magnetic stimulation of the vagus nerve

**DOI:** 10.3389/fphys.2022.938101

**Published:** 2022-10-07

**Authors:** Hongbae Jeong, Annabel Cho, Ilknur Ay, Giorgio Bonmassar

**Affiliations:** ^1^ Athinoula A. Martinos Center for Biomedical Imaging, Massachusetts General Hospital, Harvard Medical School, Charlestown, MA, United States; ^2^ Department of Bioengineering, Harvard University, Cambridge, MA, United States

**Keywords:** neuromodulation, transmission electron microscope, EM modeling, vagus nerve segmentation, k-means clustering

## Abstract

Vagus nerve stimulation (VNS) is commonly used to treat drug-resistant epilepsy and depression. The therapeutic effect of VNS depends on stimulating the afferent vagal fibers. However, the vagus is a mixed nerve containing afferent and efferent fibers, and the stimulation of cardiac efferent fibers during VNS may produce a rare but severe risk of bradyarrhythmia. This side effect is challenging to mitigate since VNS, via electrical stimulation technology used in clinical practice, requires unique electrode design and pulse optimization for selective stimulation of only the afferent fibers. Here we describe a method of VNS using micro-magnetic stimulation (µMS), which may be an alternative technique to induce a focal stimulation, enabling a selective fiber stimulation. Micro-coils were implanted into the cervical vagus nerve in adult male Wistar rats. For comparison, the physiological responses were recorded continuously before, during, and after stimulation with arterial blood pressure (ABP), respiration rate (RR), and heart rate (HR). The electrical VNS caused a decrease in ABP, RR, and HR, whereas µM-VNS only caused a transient reduction in RR. The absence of an HR modulation indicated that µM-VNS might provide an alternative technology to VNS with fewer heart-related side effects, such as bradyarrhythmia. Numerical electromagnetic simulations helped estimate the optimal coil orientation with respect to the nerve to provide information on the electric field’s spatial distribution and strength. Furthermore, a transmission emission microscope provided very high-resolution images of the cervical vagus nerve in rats, which identified two different populations of nerve fibers categorized as large and small myelinated fibers.

## 1 Introduction

Vagus Nerve Stimulation (VNS) by means of cervically implanted electrodes is an FDA-approved treatment for drug-resistant epilepsy and depression. Furthermore, VNS has become an increasingly important alternative to pharmacotherapy for other neuropsychiatric disorders (Cimpianu et al., 2016; Guiraud et al., 2016; Wang et al., 2021). The vagus is the longest nerve in the rat, consisting of 20% efferent and 80% afferent fibers (Prechtl and Powley, 1990). The typical approach with VNS is to deliver an electrical current to a pair of electrodes in galvanic contact with the left cervical vagus nerve to non-selectively target the afferent vagal fibers. These large myelinated afferent fibers, terminating in the nucleus tractus solitarius in the brainstem, are assumed to deliver the therapeutic effect through further projections into the other brain regions. However, the vagus is a mixed nerve containing afferent and efferent fibers, and unintended stimulation of the efferent fibers results in adverse effects (Howland, 2014) with a rare but severe risk of bradyarrhythmia and asystole (Åmark et al., 2007; Borusiak et al., 2009; Iriarte et al., 2009; Clark et al., 2012; Shankar et al., 2013; Schevchuck and West, 2014; Pascual, 2015; Qing et al., 2018). This cardiac side effect is challenging to mitigate since the determinant of fiber recruitment during stimulation is the fiber’s activation threshold. Thus, alternative stimulation methods, such as anodal block or high-frequency block (Tosato et al., 2007; Vuckovic et al., 2008), were studied to change fiber recruitment and directionality. Even the electric currents delivered by microscopic electrodes are diffused and can spread to undesired areas adjacent to the targeted structures, leading to unintended side effects (Histed et al., 2009; Behrend et al., 2011; Licari et al., 2011; Weitz et al., 2015). Furthermore, spatially selective VNS techniques were also studied by optimizing the pulse design and electrode design (Ordelman et al., 2013; Plachta et al., 2014; Dali et al., 2018; Aristovich et al., 2021)*.* The left branch of the vagus nerve is typically targeted since it reduces VNS side effects (Asconapé et al., 1999). However, this surgical practice is based on a single observation showing differential innervation of the canine heart by the left and the right efferent vagal fibers (Ardell and Randall, 1986). Even though different methodological approaches, such as the anodal block, have been suggested to limit non-selective stimulation, the reproducibility of the effect, mainly due to anatomical variations, is still a concern (Fitchett et al., 2021). Furthermore, patients with VNS implants experience decreased therapeutic efficacy due to scar tissue development, encasing the VNS electrodes (Aalbers et al., 2015). The metallic VNS electrodes can induce an oxidation-reduction reaction at the electrode-tissue interface, leading to an inflammatory response due to lowering the surrounding tissue’s pH (Chiu, 2012). The inflammation may lead to a reaction around the stimulation electrode track after 1 year of implantation (Navarro et al., 2007; Christensen et al., 2014). Furthermore, in some cases, VNS pulses may lead to charge accumulation (Harnack et al., 2004) and may lead to irreversible damage due to the buildup of electrolysis byproducts and thus undesired stimulation and electroporation (Crowley, 1973), which needs to be managed well in stimulus pulse design.

Microscopic Magnetic Stimulation (µMS) is an alternative way to stimulate excitable tissue (Bonmassar et al., 2012). The feasibility of using µMS to elicit neuronal activation was demonstrated *in vitro* (Bonmassar et al., 2012) and on the system level *in vivo* (Park et al., 2013). Magnetic stimulation via µMS can synaptically activate or inhibit neurons in a spatially oriented manner. Patch-clamp experiments show that, depending on the direction of the magnetic field flux, µMS generates action potentials on the axon of the ganglion cell beneath the coil (Bonmassar et al., 2012). µMS was shown to stimulate confined narrow regions (<60 μm) of cortical pyramidal neurons in brain slices *in vitro*, which helps to avoid the simultaneous activation of passing axons (Lee and Fried, 2017). µMS coils were also surgically introduced into the cochlea of anesthetized deafened felines (Blake et al., 2014), thus unresponsive to acoustic stimuli, and auditory responses were recorded during magnetic stimulation. These experiments aimed to show that the magnetic field steerability of µMS may solve low-resolution stimulation shortcomings of state-of-the-art cochlear implants. These implants are limited by their ability to reproduce accurate pitch in music and speech in the presence of background noise, which may require as much as four times the number of channels currently available (Mehta and Oxenham, 2017). Peripheral magnetic stimulation is presently used in neuropathic pain patients (Turk et al., 2008).

µMS induces a solenoidal electric field without placing the metallic micro-coil in direct contact with the tissue, generating closed-loop circular currents (Mukesh et al., 2017) and achieving a high spatial focality (Jeong et al., 2021). In µMS, no net charge is transferred from the electrode into tissue since no sinks or sources are generated when a time-varying magnetic field induces a current density. The current density in the tissue is a rotating field that mirrors the current direction in the coil. Charge balancing is a significant concern in functional electrical stimulation, as unbalanced stimulations are harmful. Any excess charge accumulation over time leads to electrolysis, resulting in electrode damage and lesions to the surrounding tissues and nerves. Both voltage and current-controlled stimulation functions (e.g., bipolar pulses) are designed to have a total null charge integrated per unit time (e.g., cycle). However, the total injected charge is not easily controllable as it depends on tissue impedance, electrode/tissue non-linearities, and electrode mismatches and may lead to a nonzero net charge (Yiğit et al., 2019). In the pulsed magnetic field case, the time-derivative of the flux of magnetic field through a surface is proportional to the electromotive force due to Lenz’s law, which is a consequence of the conservation of energy applied to electromagnetic induction Faraday’s law leading to a net null charge integrated over time. Simply, since the tissue is only excited by the time-derivative of the stimulation function, any constant term in the stimulation function is nullified by the time-derivative operation leading to a zero-mean excitation over time in the tissue (Bonmassar et al., 2014). Moreover, µMS can stimulate the brainstem nuclei in anesthetized rodents, with a net sensitivity to the directionality of the magnetic flux (Golestanirad et al., 2018).

**FIGURE 1:**
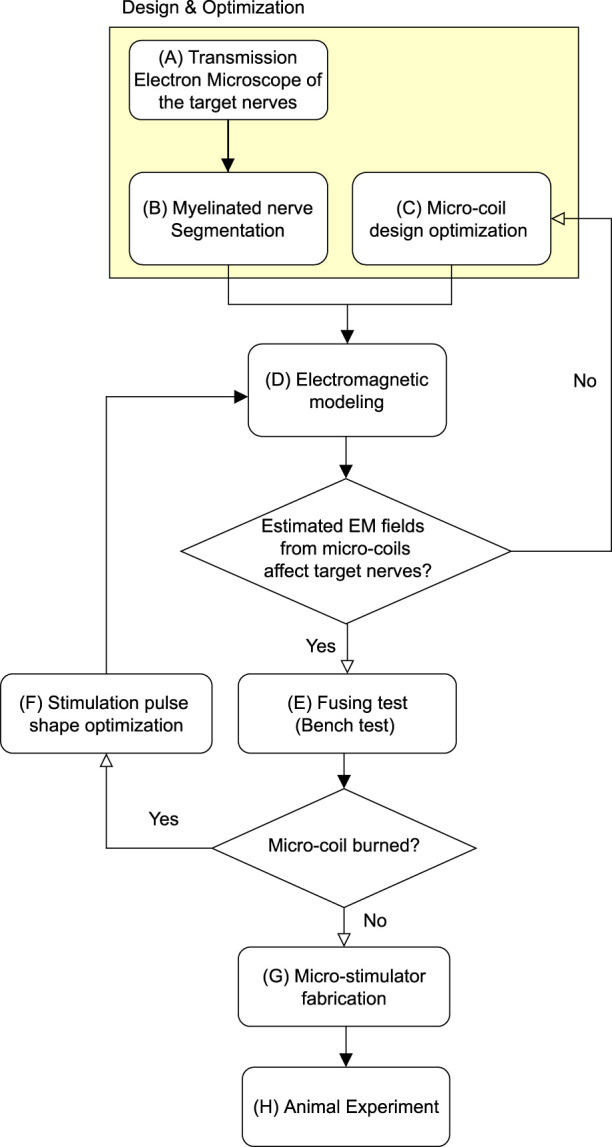
Block diagram for the proposed method. The highlighted region indicates the design and optimization stage.

The manuscript describes a novel focal stimulation of the vagus nerve using µMS. Even though µMS has been tested before (Bonmassar et al., 2012), its feasibility in VNS is presently unknown. Here we hypothesized that producing a focal time-varying magnetic field to the vagus nerve could selectively excite a targeted afferent nerve bundle, reducing the side effects while maintaining the therapeutic response. The proposed method has advantages in that it does not require direct contact of metallic electrodes with the nerve tissue as electrical VNS (eVNS), which could reduce the risk of induced RF-heating in MRI (Bonmassar and Serano, 2020) and free from charge build-up (Bonmassar et al., 2014). A transmission electron microscope (TEM) was used (Figure 1A) to investigate the myelinated fibers’ spatial distribution in the cervical vagus nerve (Fazan et al., 2001; Protasoni et al., 2009; Carvalho et al., 2013) and for medical device design (Gupta et al., 2020; Aristovich et al., 2021). Myelinated nerve segmentation (Figure 1B) was done to measure the distance of each of the large myelinated fibers to the epineurium and their clustering, as these fibers were the targets for micro-magnetic stimulation. Large fibers were clustered using the k-mean clustering algorithm (Lloyd, 1982) since multiple fibers branch to different organs, such as the heart. Based on our previous modeling results (Jeong et al., 2021), a planar spiral coil microscopic dimension was chosen (Figure 1C). Numerical studies were conducted (Figure 1D) to estimate the µMS’s optimal orientation and pulse strength. A fusing test was conducted on a benchtop, to measure the maximum pulse strength endured by the micro-stimulation coil (Figure 1E). We studied different pulse shapes (Figure 1F), which confirmed our previous study (Bonmassar et al., 2014) that exponential pulses can be optimal for minimizing coil fusing. Micro-stimulation coils were assembled for the rat vagus nerve acute stimulation and coated with special bio-compatible and dielectric polymers (Figure 1G). We examined the physiological effects (Figure 1H) of micro-magnetic VNS (µM-VNS) by monitoring respiration rate, heart rate, and arterial blood pressure in anesthetized rats (Plachta et al., 2014; Bucksot et al., 2020). Our results suggest that µM-VNS was a valuable novel technology for targeting large myelinated afferent fibers while minimizing the VNS side effects.

## 2 Methods

### 2.1 Electromagnetic simulation

**FIGURE 2:**
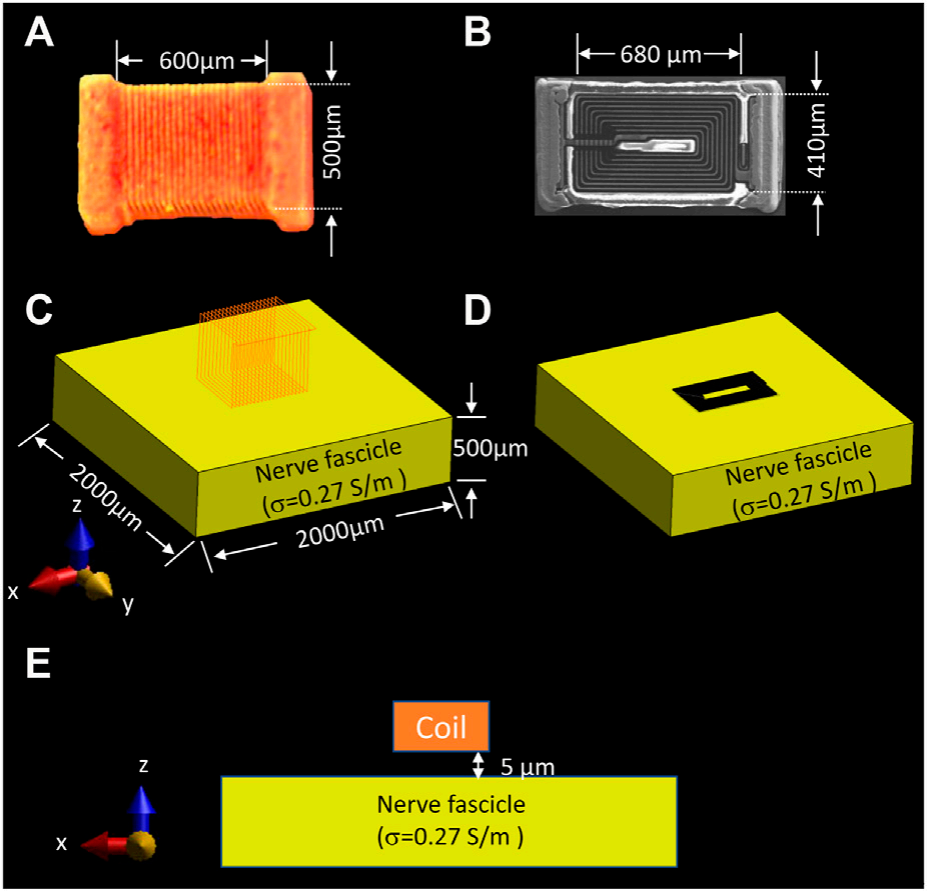
Simulation set-up for assessing the EM field behavior of two different types of coil geometries on the nerve. **(A)** dimension of the solenoidal chip inductor; **(B)** top view of the planar spiral coil scanned with Scanning Electron Microscope (SEM) and its dimension; electromagnetic simulation set-up of the **(C)** solenoidal coil, and **(D)** planar spiral coil placed on top of the nerve surface with 5 µm distance, **(E)** front view of the nerve fascicle and position of the coil. (Panel **A** was recreated from Golestanirad et al. (2018))

A numerical simulation of the electromagnetic field generated by two different coil geometries was performed to assess the electric field distribution in the rat vagus nerve, which closely matched the *in vivo* experimental set-up. The electromagnetic (EM) simulations were conducted using a quasi-static low-frequency solver in Sim4Life (Zurich, Switzerland). According to Faraday’s law of induction, a time-varying magnetic field induces an electric field (*E*) to stimulate neurons,

$$\nabla \times E=-\frac{\partial B}{\partial t}$$

where ***E*** is the electric field (V/m), and ***B*** is the magnetic field (T). The micro-inductor was chosen in this study to stimulate the nerve, where the ideal inductor could store the maximum energy in the magnetic field,

$$W=\frac{1}{2}∭J\left(x,y,z\right)∙A\left(x,y,z\right)dxdydz=\frac{1}{2}L^{*}i^{2}$$

where ***J*** is the electric current density (A/m), ***A*** is the magnetic potential (T·m), curl A is the magnetic flux density (i.e., B=∇×A ), L* is the ideal inductance (H), and *i* is the input current (A). In practice, an actual inductor has lower inductance (L∗ > L) due to magnetic field losses. That portion of energy loss is available to elicit neural activities in the µMS-VNS application. The solenoid coil (21 turns, PN ELJ-RFR10JFB, Panasonic Electronic Devices Corporation of America, Knoxville, TN, United States) and the planar spiral coil (8.5 turns, PN LQP15MN 33nH surface mount devices (SMD) inductor, Murata Manufacturing Co., Japan) was positioned 5 μm away from the nerve fiber block (σ = 0.27 S/m, εr = 5,133) to estimate the EM field generation on the nerve fiber block (Figure 2). In this study, epineurium and perineurium were not included in the simulation.

Previous studies suggested that axonal activation is ineffective when the electric field component is perpendicular to the axon direction (Amassian et al., 1989; Basser and Roth, 2000; Golestanirad et al., 2018). Thus, the activating function (AF) is the electric field gradient along the direction of the axon, which is also known as the driving function for the activation of the axon (Rattay, 1989; Ye, 2022). The AF [V/m^2^] when the nerve axon is parallel to the *x*-axis is as follows,

$$AF=\frac{\partial E_{x}}{\partial x}\;$$

The selectivity of the axon, *S*_*µM-VNS*_, was estimated,

$$S_{µM-VNS}=\frac{{VOA}_{\mu M-VNS}}{VOC}=\frac{∰\left\|{AF}_{uM-VNS}\right\|\geq 1\times 10^{5}\left[\frac{V}{m^{2}}\right]\;dx\;dy\;dz}{\pi ∙r^{2}∙h}$$

where VOA_µM-VNS_ is the volume of activation of the µM-VNS, and integrals were computed over the volume of the cylinder representing the vagus nerve [m^3^], VOC is the volume of cylindrical nerve fascicle [m^3^], ${AF}_{\mu M-VNS}$ were estimated using the minimum AF threshold of 1 × 10^5^ V/m^2^ as previously studied by Golestanirad et al. (2018), *r* is the radius of the nerve fascicle cylinder [m], and *h* is the nerve fascicle height [m].

**FIGURE 3:**
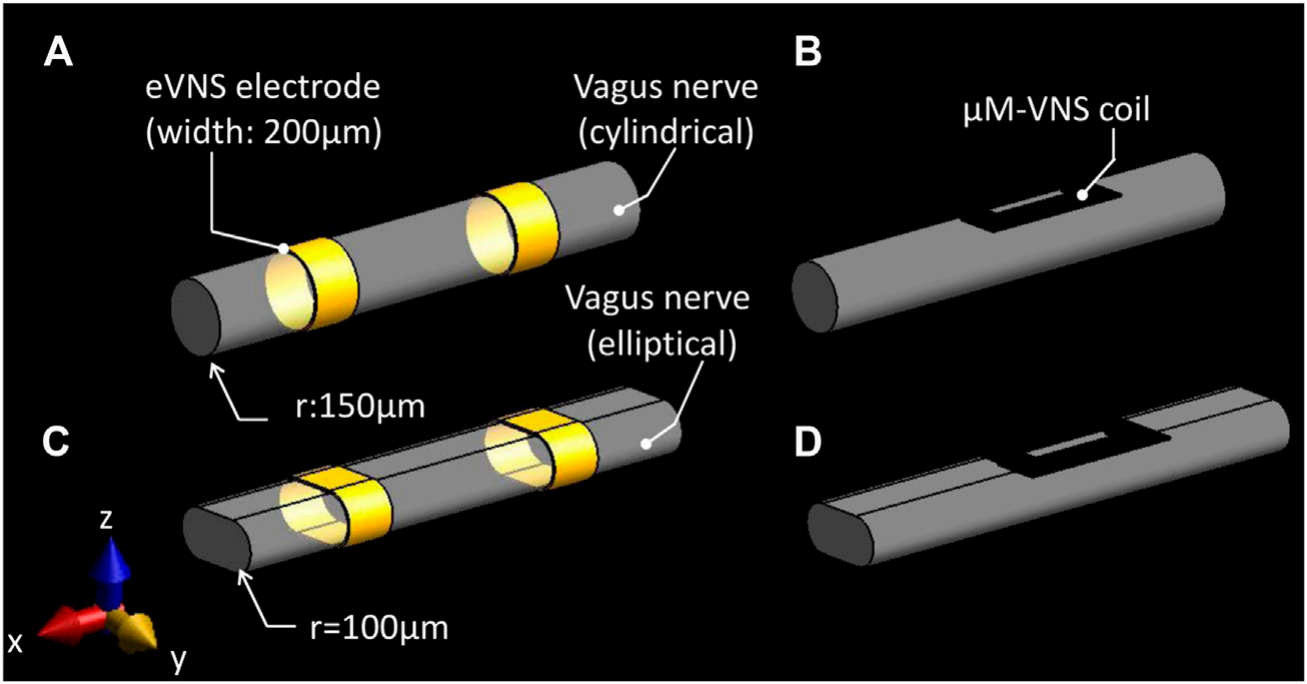
Simulation set-up for assessing two different types of vagus nerve stimulations. **(A)** eVNS using a pair of cuff electrodes wrapped around the cylindrical nerve and **(C)** flattened nerve; **(B)** µM-VNS using a planar spiral coil placed on the surface of the cylindrical nerve and **(D)** flattened nerve.

In order to compare two different types of VNS–electrical VNS (eVNS) and micro-magnetic VNS (µM-VNS)—the induced electric field (E-field) penetration was assessed using a cylindrical vagus nerve model with a radius of 150 µm (Figure 3A). A pair of cuff electrodes were wrapped around and in contact with the vagus nerve, separated by 1.5 mm. In the case of µM-VNS, the planar spiral coil was positioned 5 µm away from the vagus nerve surface (Figure 3B). Furthermore, the flattened vagus model was used to replicate the morphometric change of the nerve model by the manipulation performed during the animal experiments (Figures 3C,D).

### 2.2 Animal studies

All experiments were approved by the Massachusetts General Hospital Institutional Animal Care and Use Committee and performed in accordance with the United States Public Health Service’s Policy on Humane Care and Use of Laboratory Animals. Adult male Wistar rats (350–420 g; Charles River Laboratories, Wilmington, MA) were used to assess physiological response to the two types (electrical and magnetic) of VNS (*n* = 8) and vagus nerve segmentation (*n* = 3). For segmentation studies, only naive animals were used to avoid potential confounding effects of surgical manipulation.

a) Surgical preparation for the two types of VNS experiments.

**FIGURE 4:**
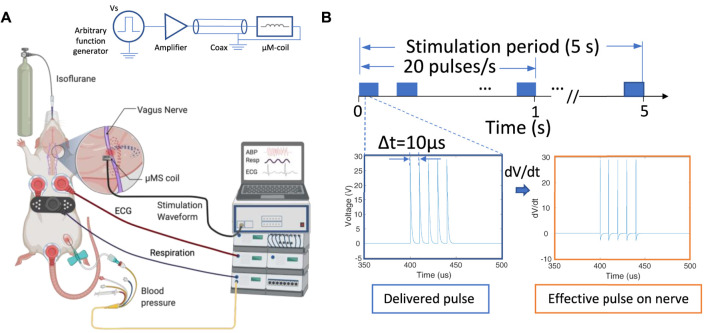
Overview of the µM-VNS experiment; **(A)** Overview and schematic of the experimental set-up; **(B)** stimulation pulses used for µM-NS that was composed of five exponential decaying pulse trains with a pulse width of 10 µs each at 20 Hz with Vp-p = 29.2 ± 3.1 V with the stimulation time for 5 s (left), and the effective pulse delivered to the nerve (right) (Figure created with http://biorender.com).

Animals were anesthetized using isoflurane (induction: 4–5% in 30% oxygen-70% nitrous oxide, maintenance: 1–2% in room air). The right femoral artery was cannulated for arterial blood pressure (ABP), a respiration sensor was placed on the abdomen to monitor the respiration rate (RR), and electrocardiogram (ECG) electrodes were placed in the Lead II position for heart rate (HR) calculations. ABP, RR, and HR were recorded continuously throughout the experiments using PowerLab 8/35 and LabChart v8.1.21 (ADInstruments, Colorado Springs, CO). eVNS electrodes and µM-VNS coil were placed on the cervical vagus nerve after separating the cervical vagus from the aortic depressor nerve and removing the epineurium/perineurium (Figure 4A), and physiological responses were recorded before, during, and after electrical and magnetic stimulation. During LabChart recordings, we used a mains filter (i.e., an adaptive filter used to remove interference related to the mains frequency - usually 50–60 Hz) built-in to LabChart with a sampling rate of 2000 samples per second. For the recording, respiration was smoothed with a triangular (Bartlett) window with 0.8-second window width. For RR, we selected a respiration belt preset with a standard deviation (s.d.) of 0.9 for peak detection. HR was calculated from ECG. We used an ECG-RAT preset for peak detection with an s.d. of 1.5.

b) Stimulation parameters.

We soldered a commercial inductor coil (PN LQP15MN 33nH SMD inductor, Murata Manufacturing Co., Japan) on a 0402 DIP adapter and connected it to an SMA connector using 36-AWG magnet wires, and fixed it inside of 1ml syringe and 16 G blunt needle (Weller, Germany). The coils were conformal vacuum coated with Parylene C, a highly biocompatible polymer with electrical insulation and water barrier properties (Merrill et al., 2005) to avoid electrical stimulation. A Parylene coating of 1 µm thickness provided electrical insulation of *R*_*insulation*_ > 100 kΩ at 100 Hz measured inside a 0.3% NaCl physiological solution between the coil and an electrode in the solution with an LCR meter (DE-5000, DER EE, New Taipei City, Taiwan). Magnetic stimulation was delivered with trains of five repeated exponential decaying pulses using an arbitrary function generator (AFG1062, Tektronix, Beaverton, OR, United States) connected to a class-D amplifier (PB717X, Pyramid, Brooklyn, NY, United States) with a bandwidth of 70kHz. A special microwave coaxial cable (PN 7029–2,555, Amphenol SV microwave, West Palm Beach, FL, United States) was selected with a low inductance to minimize magnetic flux losses along the transmission pathway between the amplifier and µM-coil (Figure 4). Two identical function generators were connected in series. The first was programmed to generate the five exponential decaying pulses (V_p-p_ = 29.3 ± 3.1 V at the output of the class D-amplifier), and the second to trigger the first function generator at 20 Hz intervals manually activated for a 5 seconds stimulation duration. As a positive control, eVNS was used. The electrode consisted of silver hook wires (0.25 mm diameter) separated 1–2 mm apart along the cervical vagus nerve (Smith et al., 2005). eVNS of 0.5 ms pulse width was delivered at 20 Hz with a stimulation duration up to 10 s with 0.5 mA using a stimulator with output isolation and constant current units (S48 stimulator, Grass Instruments, West Warwick, RI, United States), which was based on our previous studies testing the effect of eVNS in rat models of neuroprotection (Ay et al., 2011; Chen et al., 2016). The maximum current (10 A) to the coil was used to avoid the trace fuse in the given µM-VNS coil design since applying a large current has the potential to damage the traces (see Supplementary Figure S5).

### 2.3 Vagus nerve imaging and segmentation

Transmission electron microscope (TEM) imaging was used to determine the spatial distribution of myelinated fibers. The cervical vagus nerve was obtained from naive rats and was fixed in 2.5% glutaraldehyde, 3% paraformaldehyde with 5% sucrose in 0.1 M sodium cacodylate buffer (pH 7.4), and postfixed in 1% OsO_4_ in a veronal-acetate buffer. After staining *en bloc* overnight with 0.5% uranyl acetate in veronal-acetate buffer (pH 6.0), then dehydration, the nerve was embedded in Embed-812 resin (Electron Microscopy Sciences, Hatfield, PA, United States). Sections were cut on a Leica EM UC7 ultramicrotome with a Diatome diamond knife (Leica Reichert Ultracut, Bensheim, Germany) at a thickness setting of 80 nm, picked up on a mono slot grid stained with 2% uranyl acetate, and lead citrate. The sections were examined using a TEM (HT7800, Hitachi, Japan) at 85 kV and photographed with an advanced microscopy techniques (AMT) charge-coupled device (CCD) camera. The images were preprocessed for noise removal, stitched (Panorama Stitcher), and segmented using MATLAB (MathWorks, MA, United States). The original TEM images consisted of multiple scans of the whole vagus nerve and were then stitched together. For segmentation, the images were adjusted for contrast, masked based on contrast thresholds, and partitioned based on three main layers of the masks. An image “*particle analysis*” tool in ImageJ (NIH, Bethesda, MD, United States) was used, and it was based on the grayscale threshold to determine each fiber’s and myelinated fibers’ diameters and locations. Large myelinated fibers were defined as those with a diameter larger than the average median of the myelinated fiber diameter among three rats, whereas small myelinated fibers were determined to be myelinated fibers with a diameter smaller than the median average of myelinated fibers. Unmyelinated fibers were excluded from the analysis. (see Supplementary Figure S1 for the detailed segmentation process flow chart).

### 2.4 Temperature safety benchtop study

The fiber optic probe measured thermal elevation on the µM-VNS on the bench (OSENSA Innovations Corp., Coquitlam, BC, Canada). Two-channel probes were used to measure the devices’ heating. The first was placed on the surface of the active coil (i.e., with the supplied stimulation pulses), while the second channel was positioned on the surface of the control coil (i.e., without stimulation pulses). The identical pulses used in the μM-VNS experiments presented above were also used in the temperature studies.

### 2.5 Data analysis

Python (Van Rossum and Drake Jr, 1995) and Prism8 (GraphPad, San Diego, CA, United States) software were used for quantitative distribution and statistical analysis (see Supplementary Code S1).

The clustering algorithm was based on the MATLAB script, which selected only the fiber of a diameter greater than 2.97 µm in an automated calculation. The location of these large myelinated fibers was then fed into k-means clustering algorithm, which automatically produced the clustering Figure 11. K-means clustering method (Lloyd, 1982) was used to cluster the larger myelinated fibers with distance measure using the sum of absolute differences (i.e., *citiblock*) for five repetitions in eleven clusters using the “*k-means clustering function*” in MATLAB (see Supplementary Code S2). The cluster number was chosen empirically for each rat, as each cluster contained the maximum number of comparable size fibers, while the results were not well clustered for different cluster numbers. Continuous physiological variables were averaged before and during the stimulation for 5 s. Data were expressed as mean ± standard error of the mean. The normality of the data was tested using the *Shapiro-Wilk test.* Comparisons within groups were performed using a 2-way analysis of variance (ANOVA) followed by Tukey’s multiple comparison test when needed for data that showed normal distribution. *Multiple Wilcoxon test* was used when the data were not normally distributed. *P* values less than 0.05 were considered significant. The results of the respective test method were presented in the Results section.

## 3 Results

### 3.1 Electromagnetic simulation

**FIGURE 5:**
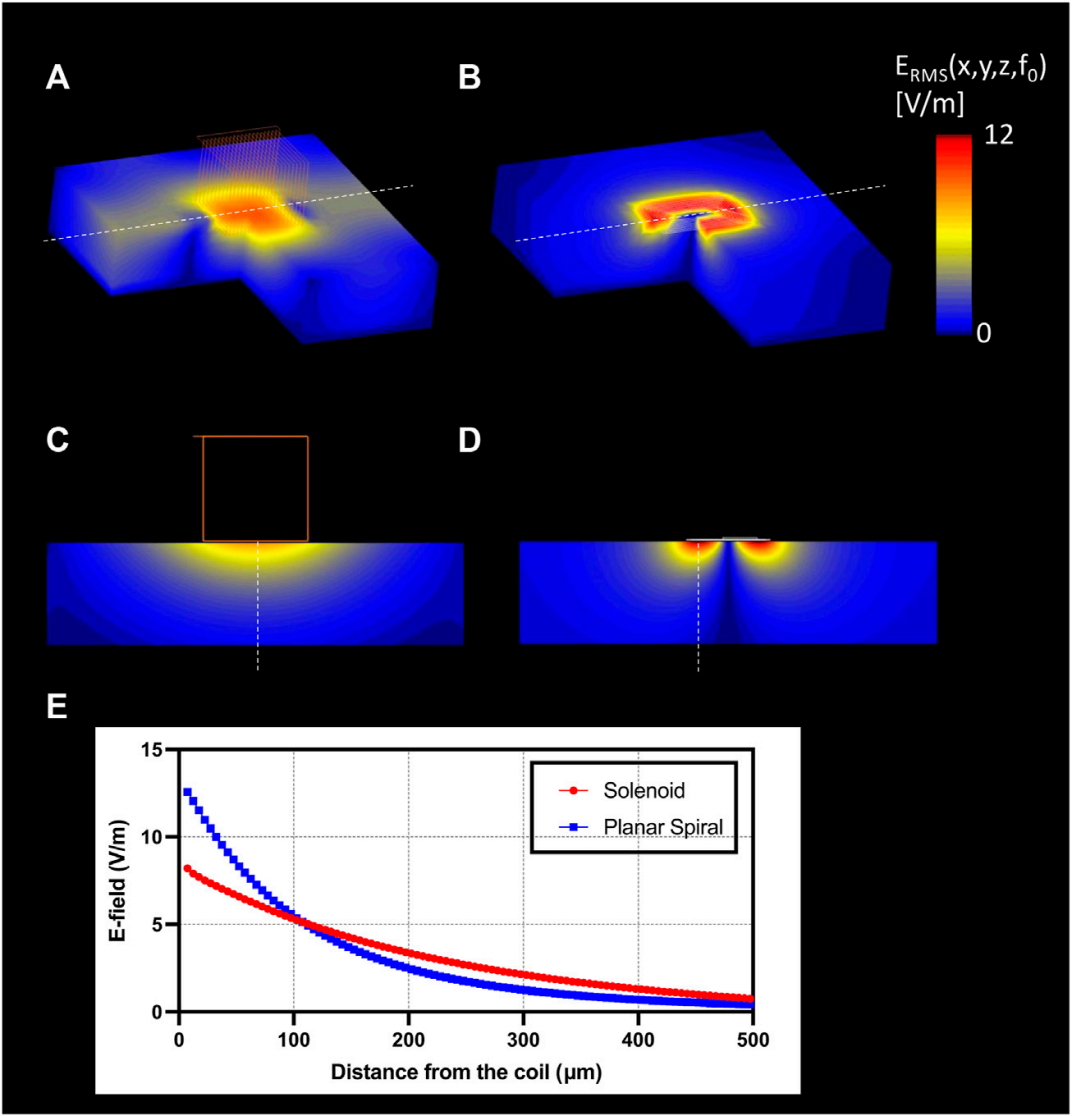
EM simulation results. 3D surface view of the E-field generation on the nerve with **(A)** solenoidal coil (21 turns, wire diameter: 7 µm); **(B)** planar spiral coil (8.5 turns, trace width: 7 µm); Slice view of the E-field penetration in the central view with **(C)** solenoid coil, **(D)** planar spiral coil; **(E)** profile of the |E|RMS (blue), and gradient of |E|RMS (red) along with the maximum E-field projection (red: solenoid coil, blue: planar spiral coil).

**FIGURE 6:**
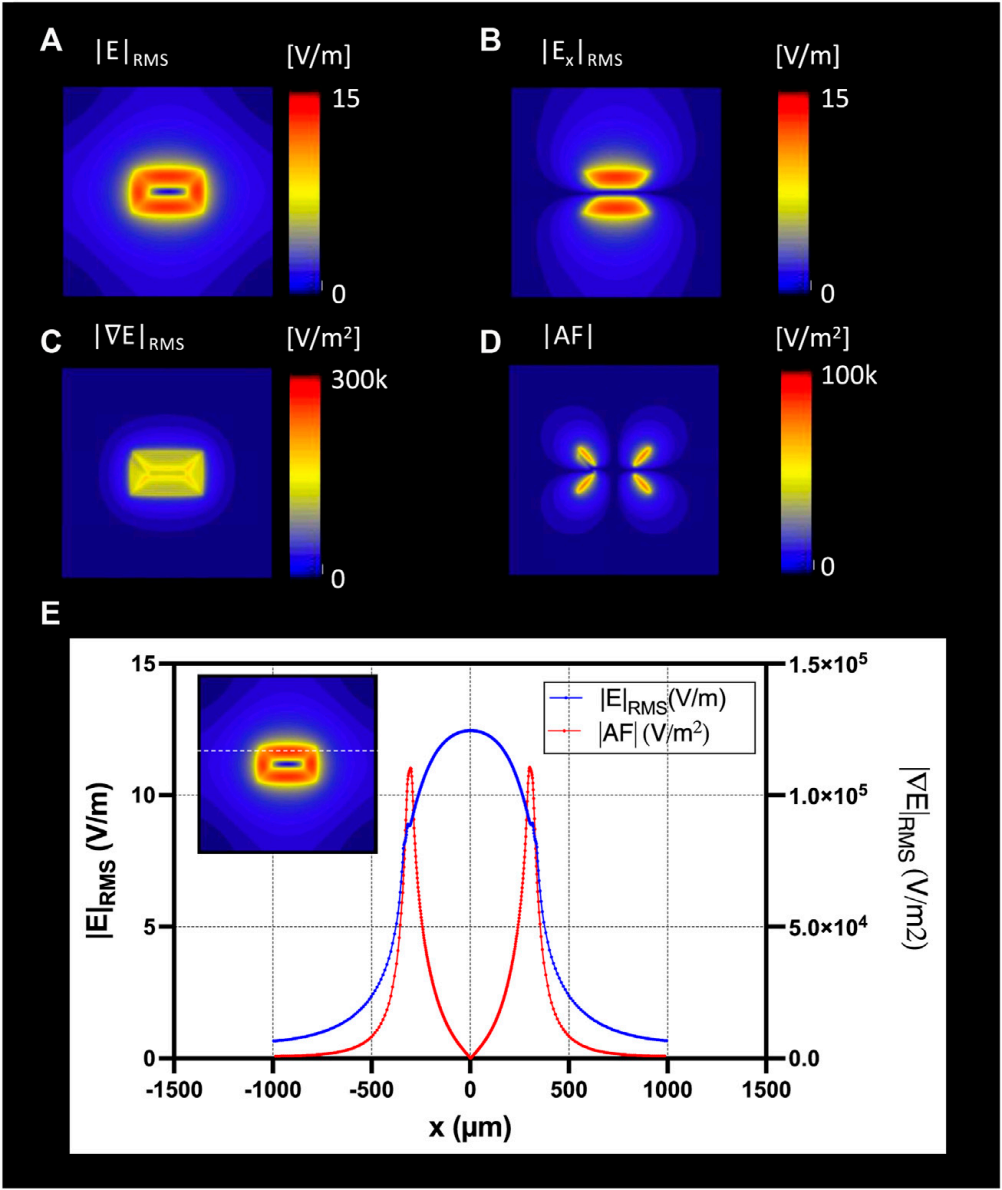
The optimal coil orientation was along the *x*-axis; Top view of the E-field generation on the nerve surface with **(A)** |E|RMS; **(B)** |Ex|RMS; **(C)** gradient of ERMS; **(D)** Top view of the absolute Activating Fucntion (AF): spatial gradient of Ex in *x*-direction; **(E)** profile of the |E|RMS (blue), and gradient of |E|RMS (red) along the nerve direction along the *x*-axis.

Electromagnetic (EM) simulations were performed to optimize the overall shape of the micro-stimulation coil. Figure 5 shows that the planar spiral coil provides a 1.53 times increase in the root mean square (RMS) electric field (E-field) than a solenoidal coil on the nerve surface in solenoid geometry. The planar spiral coil was superior to the solenoidal coil at depths up to 110 µm inside the nerve (Figure 5E). EM simulation was also performed to find the optimal orientation and the position of the micro-stimulation coil with respect to the axis of the fibers. We found that the ideal coil orientation and position (Figure 6G) was centering a coil on the nerve axis with the longest side parallel to the axis. Finally, EM simulation was performed to establish the amount of the current needed to generate at least 6 V/m since it was shown in previous studies (Golestanirad et al., 2018) to be a threshold for the activation of large myelinated fibers. The simulation estimated that the pulse with current peaks of 10 A induced an RMS electric field (E-field) of at least 6 V/m at a depth of 87.5 µm (Figure 6G) and the peak RMS gradient |E|-field of 135.6 k V/m^2^ at 275 µm from the center of the coil (Figures 6D,G).

Figure 6 shows the RMS estimated E-field in the x-directions on a nerve fascicle block surface 5 μm under the µM-VNS coil with spatial partial derivatives (see Supplementary Figure S2 with all nine partial derivatives). The results of EM simulations showed, with seven turns and 10 A, a maximum RMS E-field strength of 12.7 V/m in the nerve and a maximum |AF| of 1.6 × 10^5^ V/m^2^ (Figure 6G).

**FIGURE 7:**
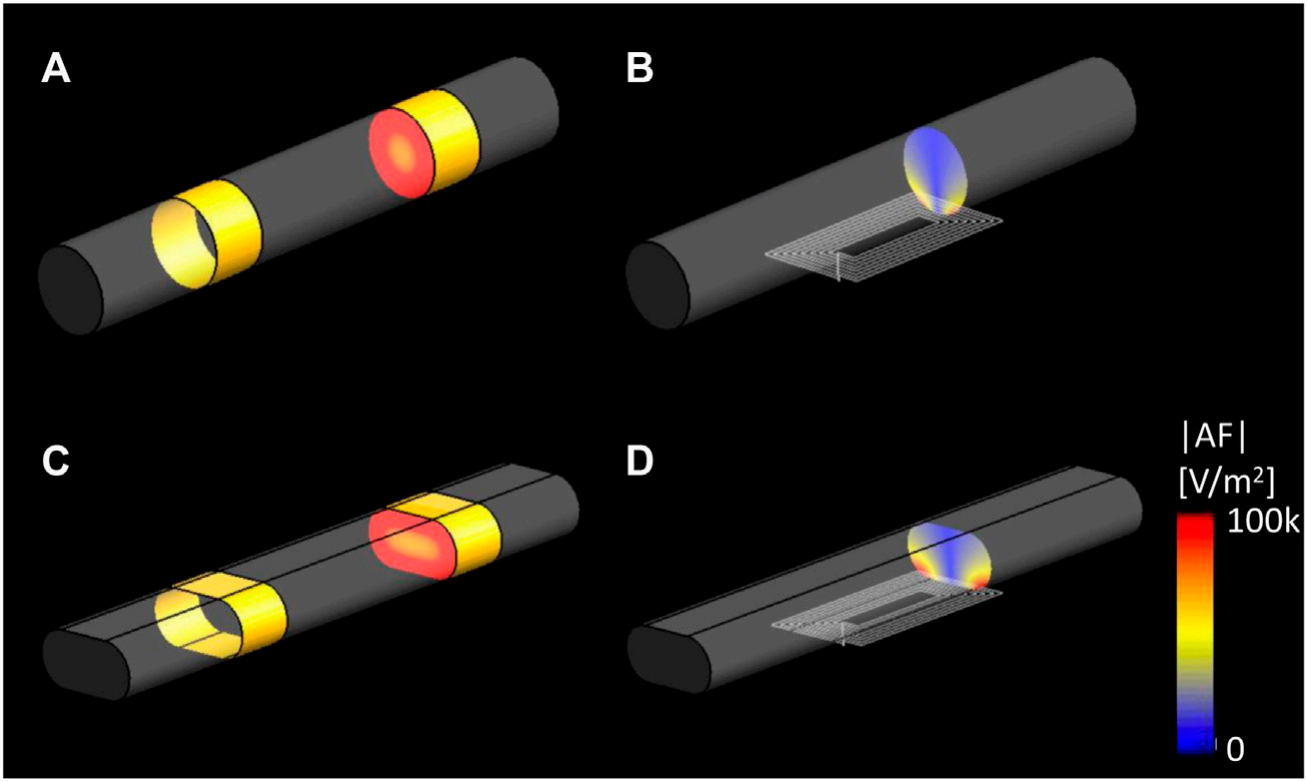
EM simulation results of |E|RMS on the cylindrical and elliptical nerve. Cross-sectioned view of the |AF|, **(A)** eVNS on the cylindrical nerve and **(C)** flattened nerve; **(B)** µM-VNS on the cylindrical nerve and **(D)** flattened nerve.

Figures 7A,C show that in the cylindrical and flattened nerve cases, the electrical VNS (eVNS) induced a uniform E-field and AF in the nerve with the highest |E|-field RMS values of 35.22 V/m and 36.68 V/m, and the highest absolute AF values of 1.10 × 10^7^ V/m^2^, and 2.82 × 10^5^ V/m^2^, respectively. In contrast, the µM-VNS induced a focal E-field in the nerve with the highest |E|-field RMS of 11.10 V/m and 16.14 V/m for the cylindrical and flattened nerve models and maximum absolute AF values 1.25 × 10^5^ V/m^2^, and 2.82 × 10^5^ V/m^2^, respectively (Figures 7B,D). The selectivity, S_µM-VNS_, in the cylindrical and flattened nerve cases was calculated as 7.07 × 10^−4^ and 7.00 × 10^−4^, respectively.

### 3.2 Animal studies

None of the animals died or experienced complications during the electrode implantation or stimulation-recording period.

**FIGURE 8:**
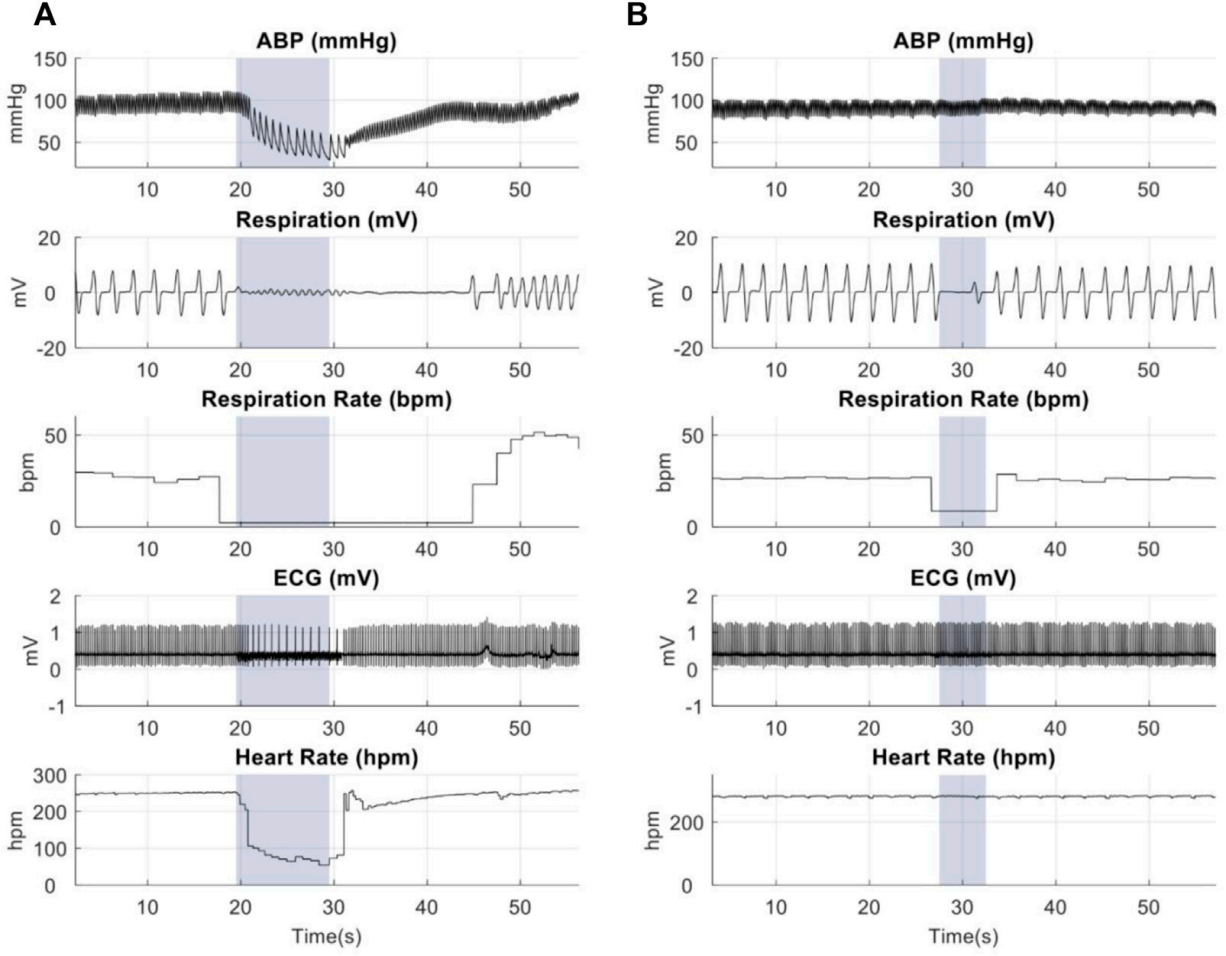
Representative physiological recordings in animals receiving **(A)** eVNS (0.5 mA, 0.5 ms, 20 Hz), and **(B)** µM-VNS (Vp-p = 29.2 ± 3.1 V, 20 Hz). The blue box shows the stimulation period.

Animal studies were performed to confirm the focality of the µMS by testing the hypothesis that the focal stimulation of the vagus nerve produces fewer side effects. Microscopic magnetic stimulation was performed on the vagus nerve to affect only one physiological response to the neurostimulation without affecting the others (e.g., changing the respiration rate (RR) without affecting the heart rate (HR)). Physiological responses to eVNS in an individual animal are shown in Figure 8A. The eVNS induced a transient 32.4 ± 12.8% reduction in arterial blood pressure (ABP) that gradually recovered after 12 s. Similarly, RR and HR exhibited 77.0 ± 11.0% and 55.3 ± 18.8% (average of all rats, *n* = 8) reduction during the stimulation. The physiological responses of µM-VNS are shown in Figure 8B. The µM-VNS induced a non-significant decrease of 0.3 ± 2.4% in ABP for the duration of the stimulation. In contrast, only RR exhibited a decrease of 50.1 ± 16.2% during the stimulation period, while HR showed a non-significant decrease of 0.3 ± 0.5% (average of all rats, *n* = 5) during stimulation. (for further details, please see Supplementary Table S1).

**FIGURE 9:**
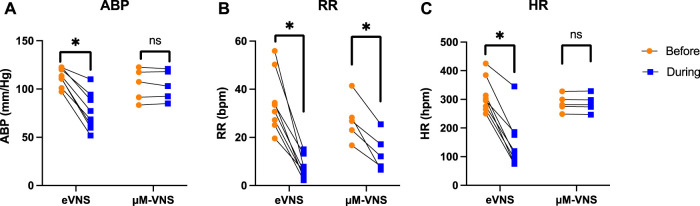
Electrical stimulation (*n* = 8) decreases **(A)** arterial blood pressure (ABP), **(B)** respiration rate (RR), and **(C)** heart rate (HR), whereas µM-VNS (*n* = 5) provides a more selective effect by decreasing only the RR. * *p* < 0.05, ns: non-significant (for further details, please see Supplementary Table S1).

Overall effects of eVNS and µM-VNS on ABP, RR, and HR are shown in Figure 9. There was a significant ABP, RR, and HR decrease after eVNS in all animals (*n* = 8), indicating a non-selective stimulation of afferent and efferent fibers. In contrast, µM-VNS only charged physiological parameters in some rats (5 out of 8). Moreover, the µM-VNS-induced effect was limited to RR without affecting ABP and HR, suggesting a more selective vagal fiber stimulation. For statistical analysis, see Supplementary Information S5.

### 3.3 Pulse shape optimization and fusing test

Exponential pulses were used as they were found ideal in a previous study (Bonmassar et al., 2014). In order to confirm the efficacy of the new short pulses µMS, we have also conducted sciatic nerve stimulation studies that supported the 10 µs efficacy of five exponential pulses of magnetic stimulation (Supplementary Figure S3). Magnetic stimulation could activate the muscles by magnetically and electrically stimulating the sciatic nerve with the same experimental parameters used for micromagnetic VNS (µM-VNS) and eVNS (Supplementary Figure S4). The exponentially decaying pulse was used as they were found ideal in a previous study (Bonmassar et al., 2014), reducing the delivered power level while maintaining the amplitude of the time-varying magnetic field. Our benchtop results (see Supplementary Figure S5, and Supplementary Figure S7) showed that the coils fused only for currents greater than 10A, thus acting as a fast fuse (Supplementary Figure S6). The fusing threshold using similar square pulses was found to be much lower but was never precisely estimated because it required loss of coils.

### 3.4 Pulse shape optimization and fusing test

Exponential pulses were used as they were found ideal in a previous study (Bonmassar et al., 2014). In order to confirm the efficacy of the new short pulses µMS, we have also conducted sciatic nerve stimulation studies that supported the 10 µs efficacy of five exponential pulses of magnetic stimulation (Supplementary Figure S3). Magnetic stimulation could activate the muscles by magnetically and electrically stimulating the sciatic nerve with the same experimental parameters used for micro magnetic VNS (µM-VNS) and eVNS (Supplementary Figure S4). The exponentially decaying pulse was used as they were found ideal in a previous study (Bonmassar et al., 2014), reducing the delivered power level while maintaining the amplitude of the time-varying magnetic field. Our benchtop results (see Supplementary Figure S5, and Supplementary Figure S7) showed that the coils fused only for currents greater than 10A, thus acting as a fast fuse (Supplementary Figure S6). The fusing threshold using similar square pulses was found to be much lower but was never precisely estimated because it required loss of coils.

### 3.5 Cervical vagus nerve segmentation

**FIGURE 10:**
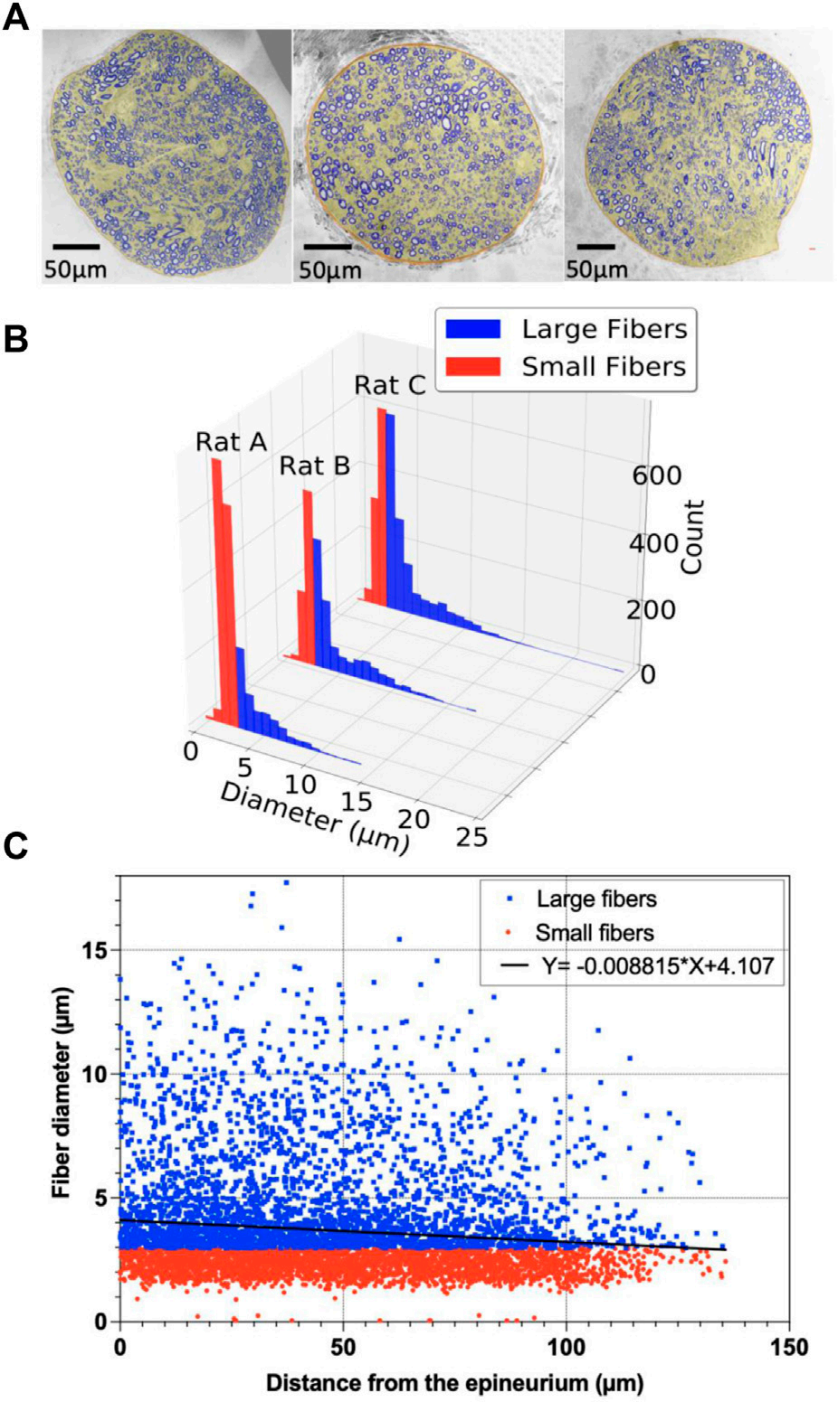
Segmentation of the vagus nerve; **(A)** Stitched transmission electron microscope (TEM) image (magnification: ×300) of the cervical vagus nerve of adult Wistar rats and MATLAB segmented vagus nerve overlay on the top; **(B)** Histogram of large and small fibers for rats A**–**C; **(C)** Scatter plot of the myelinated fibers with the diameter (µm) as a function of distance (µm) from the epineurium of the vagus nerve for rats A–C, and the point estimate of the slope of the regression model.

A transmission emission microscope (TEM) was performed to understand the histology of the rat vagus nerve and to cover the spatial distribution of the large myelinated fibers and clustering. In order to achieve this goal, segmentation was performed to locate the large myelinated fibers. Figure 10 shows the vagus nerve segmentation results of three rats. The vagus nerves had an estimated average diameter of 318.17 ± 60.67 μm. The average median diameter of the myelinated fibers was 2.98 µm (for more details, see Table 1).

**TABLE 1:** Morphometric parameters of the segments of the vagus nerve in three rats.

	Rat 1	Rat 2	Rat 3
Nerve Area (μm^2^)	51,699.28	80,358.72	112,119.05
Nerve diameter (μm)	256.63	319.95	377.92
Myelinated fiber number	2,126	1795	2,397
Myelinated fiber density (10^3^ fibers/mm^2^)	41.12	22.32	21.38
Median of myelinated fiber diameter	2.49	3.21	3.23
Large-fiber number	672	1,054	1,439
Large-fiber density (fiber/mm^2^)	12,998	13,116	12,835

The linear regression of the myelinated fiber diameter versus the distance from the epineurium of the nerve is shown in Figure 10C. The point estimate of the regression slope was -0.009, and the 95% confidence intervals (CI) were [-0.007 to -0.011] and did not include 0 (Figure 10C).

**FIGURE 11:**
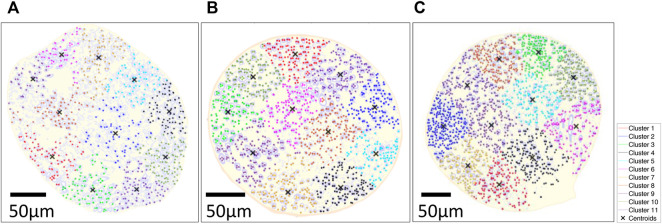
k-means clustered on large fiber group in large fiber group overlaid on the segmented results of three rats; **(A)** Rat A; **(B)** Rat B; **(C)** Rat **(C)** k-means clustering algorithm was used with distance measure using the sum of absolute differences (i.e., citiblock) as a cost function with the five repetitions with 11 clusters displayed in different colormap and 'X' as centroids of each cluster. The fiber diameter was used for weight on k-means cluster (i.e., w = 1: 3–5 µm, w = 2: 6–9 µm, w = 3: 9–12 µm, w = 4: 12–15 µm, w = 5: >15 µm).

The k-means clustered large fiber group (fiber diameter ≥2.98 µm) is shown in Figure 11. Each rat had two center clusters, while the remaining were next to the epineurium. The cluster number was chosen empirically to be k = 11 for each rat, as each cluster contained the maximum number of comparable size fibers since the k-means results were not well clustered for other cluster numbers (e.g., k = 6 or k = 16, etc.).

### 3.6 Temperature safety benchtop study

**FIGURE 12:**
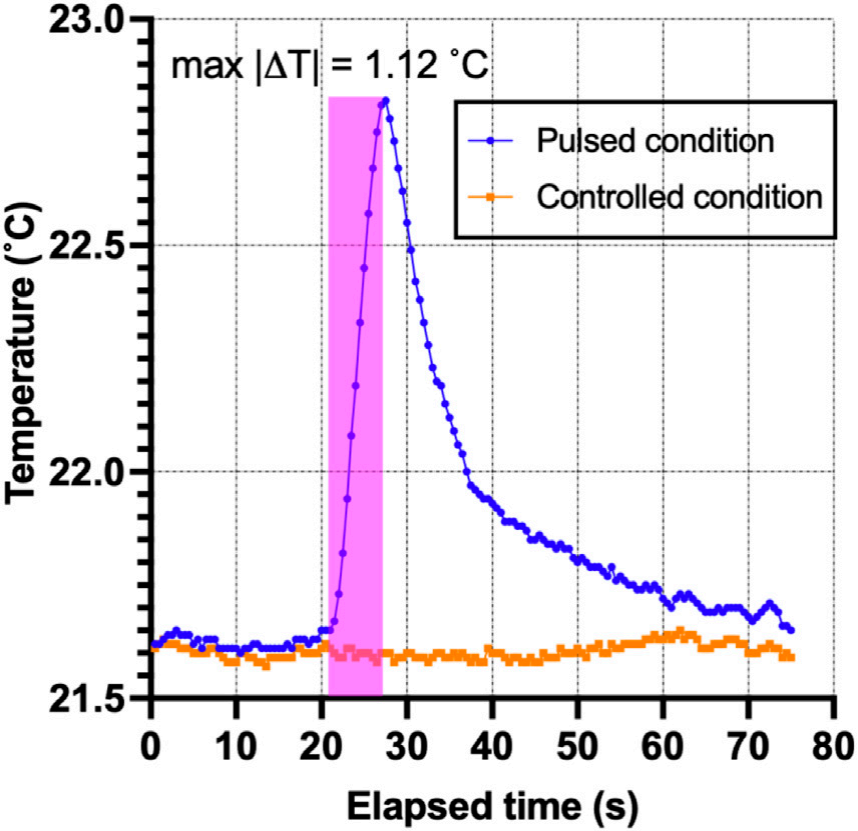
Thermal elevation experiment. The fiber-optic probe measured the point temperature changes in the µMS for the pulsed and control conditions over time.

Safety temperature measurements were performed to ensure that the micro-stimulation coils would not thermally damage the vagus nerve of the rats. The optic probe was positioned on the µM coil measurement over 5 s using the same stimulation trains applied in the animal studies (Figure 4), resulting in only a minimal thermal elevation of 1.12°C during the magnetic stimulation shown as a blue trace in Figure 12. The orange trace corresponds to the heating in the control condition, which does not show any temperature elevation as expected since no pulses were delivered to the coil.

## 4 Discussion

We employed an emerging technology, micro-magnetic stimulation, to stimulate the cervical vagus nerve. Micro-magnetic VNS (µM-VNS) was used to focally stimulate the vagus nerve while minimizing efferent fiber stimulation and the occurrence of bradycardia. The prototype coil used to stimulate single clustered fibers has a planar spiral trace geometry capable of carrying large current pulses to elicit neural activation via electromagnetic (EM) induction. Furthermore, as the quasi-static EM simulations show, µM-VNS induces electric fields with a high spatial gradient due to their microscopic dimension. Lastly, µM-VNS can help address other technological risk arising from electrical VNS (eVNS), such as induced RF-heating that are only allowed to be used in labeled conditions such as outside of local transmit coil coverage and low-powered pulse sequences (Shellock et al., 2006), as previously shown and tested with clinically allowed worst-case condition (Bonmassar and Serano, 2020).

Golestanirad et al. (2018) showed that a lower E-field strength of 6 V/m and a lower spatial electric field gradient of 1 × 10^5^ V/m^2^ results in neural activation of fibers using a Neuron Model (Johnson and McIntyre, 2008) with 20A and 20 turns. Unlike Golestanirad et al., we used a planar spiral coil since the EM simulations indicated that this geometry, compared to a solenoidal one, could induce a higher E-field within 110 µm inside the nerve (Figures 5, 6). Furthermore, Lee et al. (2016) showed that magnetic neurostimulation could be achieved with a much lower E-field strength of 2.1 V/m but with a high electric field gradient of 5.0 × 10^4^ V/m^2^ using a micro-fabricated coil with only 1 mA and a single turn. However, these fields were estimated and reported on the tip of the coil and not inside the tissue. A figure-of-8 geometry could be used to augment the resulting E-field strength, and our previous work demonstrated that the figure-of-8 coil geometry could generate the enhanced E-field (Jeong et al., 2021).

These quasi-static EM solver simulation results were confirmed in rat physiological response studies. During these *in vivo* experiments, µM-VNS pulse trains were used to stimulate the cervical vagus nerve bundles, resulting in 62.5% of the animals (5 out of 8) having a decreased respiration rate (RR) while none of the animals had any change in heart rate (HR) and arterial blood pressure (ABP). Conversely, eVNS in full recruitment (simplified worst-case scenario) caused a decreased RR, HR, and ABP in all animals (8 out of 8). This different physiological response was likely caused by the magnetic stimulation activating only a few clusters of fibers. In contrast, the electrical stimulation condition caused a non-selective fiber activation as an example of the worst-case scenario of full recruitment. A possible explanation is that in the subset of responding animals, the µM-VNS stimulated the afferent fibers that project into nuclei of the brain that control respiration. These results are in line with Ahmed *et al.'s* rostral and caudal vagotomy study in rats (Ahmed et al., 2020), which demonstrated that the decrease in RR as an afferent effect (i.e., observed after caudal vagotomy), whereas HR reduction as an efferent stimulation effect (i.e., observed after rostral vagotomy). In contrast to the previous studies, we omitted the epineurium and perineurium of the rat vagus nerve in our modeling (Bucksot et al., 2019) because we surgically removed the epineurium and perineurium of the nerve prior to µM-VNS.

Longer pulse widths (100 µs or more) can reduce the nerve stimulation threshold (Alon et al., 1983; Gorman and Mortimer, 1983; Reilly, 1998). Even though eVNS commonly employs 200 µs or more pulse widths, ultra-short pulse widths were also investigated. Rattay et al. (2012), investigated chronaxies of pyramidal cells with axonal pulse duration down to 10 µs. Furthermore, Peterchev *et al.*, using transcranial magnetic stimulation, showed stimulation with 20 µs pulse width (Peterchev et al., 2008). Electromyography (EMG) was used to test and optimize the stimulation pulse shape in the rats’ sciatic nerves. Similar to our approach, Kagan et al. (2016) also showed EMG responses during sciatic nerve magnetic stimulation.

Transmission Emission Microscope (TEM) (i.e., pixel size: 27.3 nm) images of the rat cervical vagus nerve were used to quantify the statistical distribution of the target large, myelinated fibers and to help interpret the results of the rodent studies. Large fibers of the vagus nerves of spontaneously hypertensive rats are clustered in peripheral regions of the vagus nerve (Licursi de Alcântara et al., 2008). Our study showed a similar spatial histological distribution in healthy Wistar rats, and we have presented a comprehensible way of representing such clusters using k-means clustering (Lloyd, 1982). K-mean cluster results were calculated with large myelinated fibers’ location and diameter as input parameters, thus providing the additional information from the histogram shown in Figure 10B, which solely shows the two populations of fiber size without spatial information. To determine the number of clusters, we did myelinated fiber segmentation and the k-mean cluster algorithm to understand the spatial information. Eleven clusters were identified visually to help readers visualize the cluster organization of the fibers in the vagus nerve. The vagus nerve branches to different organs (e.g., heart, lungs, stomach, intestine, etc.). In future work, we will investigate the relationships between the clusters and the various branches of the vagus nerve.

Benchtop thermal measurements (Figure 11) showed that the thermal elevation during the stimulation pulse was less than 1.2°C, below the thermal damage of neural tissue (Yarmolenko et al., 2011; van Rhoon et al., 2013) and neurostimulation (Chen et al., 2015) thresholds.

### 4.1 Limitations

The study was designed to target only proximally located afferent fibers, albeit not directly detectable with TEM. However, the statistical distribution of the diameter of the myelinated fibers over space revealed two distinct populations: the small and large myelinated fibers. One may assume that these were the afferent and efferent fiber populations, but at present, we do not have any direct evidence to confirm this hypothesis, and further studies need to be completed. In order to differentiate between afferent and efferent fibers, there are two different methods: neuronal tracing (Walter et al., 2009; Zheng et al., 2022) and conduction velocity measurements (McAllen et al., 2018).

Given the hypothesis of focal stimulation of the micro-stimulation coil, we did not expect to observe RR, HR, and ABP responses in all rats. The vagus nerve projects not only to the brain, lungs, and heart but also to many other organs in the body. If we did see a stimulation in 100% of the rats, it meant that the micro-magnetic stimulation was not focal. Fortunately, we could not localize and stimulate the afferent fibers in all acute animal preparations, and this blind targeting resulted in 37.5% non-responsive rats in the µM-VNS studies. Conversely, the eVNS studies did not produce non-responsive rats since the stimulation was performed indiscriminately over the entire vagus nerve. A limitation of the study was that we did not have the means to establish that other fibers were stimulated in non-responsive rats since we only measured RR, HR, and ABP. A µM-VNS array could provide targeted stimulation (Plachta et al., 2014; Aristovich et al., 2021), which could be performed during neurostimulation programming to maximize the therapeutic while minimizing the side effects.

Contrary to humans, the anatomy of the cervical vagus nerve does not differ between the left and right branches (Licursi de Alcântara et al., 2008), and no significant differences in stimulation responses were reported in rat studies (Ay et al., 2011; Strauss et al., 2019; Bucksot et al., 2020). Thus, the left and right cervical vagus nerves were not studied separately. This study only investigated the physiological response by magnetic stimulation on the cervical vagus nerve in the rat, thus may not directly indicate any therapeutic effects. Additionally, the studies were conducted under isoflurane. Therefore any generalization should take that into account, especially since it is established that the threshold to induce physiological responses is higher under anesthesia (Ahmed et al., 2021).

The mechanical stress on the vagus nerve may also produce scar tissue in the Vagus Nerve, which could lead to unwanted axon coil distance in the case of µM-VNS. Further studies are needed to test mechanical stress against µM-VNS coil implantation. We did not study the realistic pathway of the nerve fibers in our simulations. With our slicing and dicing technique, we couldn’t acquire serial sections with our TEM (HT7800, Hitachi, Japan) that require manual sectioning and registration of the multiple sections. The success rate of the manual sectioning and staining did not return any consistent data that could be used for the three-dimensional modeling of the vagus nerve fiber distribution. In future studies, we plan to use serial section scanning electron microscopy of rats’ vagus nerve tissue using focused ion-beam milling (Xu et al., 2021). Also, we only tested the inner fascicle of the vagus nerve in rats.

Finally, as reported before (Ahmed et al., 2020), the observed decrease in RR may be due to the afferent vagus nerve stimulation. However, further studies are necessary to understand this mechanism better, which could be performed either by using functional magnetic resonance imaging (Cao et al., 2017) or evoked compound action potentials (Mollet et al., 2013).

Despite the potential advantages of µMS, its current energy consumption is its weakness as an implantable device. In our experiment, µM-VNS required 51.8 mJ (e.g., exponential pulse with an efficiency of 0.16, peak-voltage (V_peak_): 32.4V with 10A maximum input current, five pulses at 10 µs at 20 Hz), whereas eVNS required 2.5 µJ (1kΩ resistance with 0.5 mA input current for 0.5 ms pulse width at 20 Hz). Furthermore, we were not able to study µMS response with greater intensities and pulse durations due to limitations in the thin film construction of Murata’s surface mount inductor, which resulted in fusing (see Supplementary Information S4). In future work, we plan to create a custom-made microscopic magnetic stimulation device (Jeong et al., 2021) that will address this limitation. Finally, we were not able to rotate the µM-VNS coil around the vagus nerve because the current surface mount device could only be positioned underneath the vagus nerve. The nerve had to be stretched on top of the coil (see Supplementary Figure S9), and it is impossible to rotate the nerve without mechanical stimulation of the vagal fibers since the nerve enters the thoracic cavity on the distal end and dives deep into the neck to enter the cranium on the proximal.

## 5 Conclusion

This study found that short-pulse magnetic vagus nerve stimulation might reduce side effects by targeting a peripheral and proximal subset of myelinated fibers in rats. The eVNS caused a decrease in ABP, RR, and HR, whereas µM-VNS only caused a transient reduction in RR. The absence of an HR response indicated that µM-VNS might provide an alternative technology to eVNS with a reduced risk of RF-induced heating in MRI (Bonmassar and Serano, 2020), albeit referenced study was done for deep brain stimulation. Numerical EM simulation estimated the strength and spatial distribution of the electric field in the cervical vagus nerve and suggested an optimal coil orientation profile. Benchtop heating measurement confirmed that the thermal elevation of the µM-VNS was lower than the threshold to damage the nerve tissue and stimulate the nerve by thermal elevation. Finally, the TEM studies revealed the presence of two distinct myelinated fiber populations (i.e., large and small fibers) and their spatial distribution. There were only two clusters in the center, while the remaining nine were all located along the epineurium, which allows for peripheral targeting. VNS using µMS is a novel approach that could yield similar spatially focal stimulation as multi-contact eVNS electrodes with reduced risk of RF-induced heating in MRI. Given the benefit of focality that we observed (i.e., minimal unintended effects like a reduction in heart rate), future work will investigate the brain responses to µM-VNS to confirm its success in activating the fibers responsible for the therapeutic effects of VNS. These future studies will increase understanding of the neuroscience of VNS by establishing the link between cervical vagal fibers (including their topographical location in the nerve) and their connections in the brainstem. In addition, contingent on developing more compact magnetic stimulus generators, this study could provide alternative therapeutic approaches to patients that may benefit from VNS (e.g., Alzheimer’s disease, traumatic brain injury, inflammatory bowel syndrome, stroke, epilepsy, and depression) (Johnson and Wilson, 2018; Wang et al., 2021).

## Data Availability

The raw data supporting the conclusions of this article will be made available by the authors, without undue reservation.
