# Supplementary material for: Short-pulsed micro-magnetic stimulation of the vagus nerve
Source: Front Physiol. 2022 Oct 7;13:938101. doi: 10.3389/fphys.2022.938101 (PMC9585240; doi:10.3389/fphys.2022.938101)
Supplement: Supplementary file 1 [file DataSheet1.pdf]

<u>Supporting Information:</u>

Short-pulsed micro-magnetic stimulation of the vagus nerve

Hongbae Jeong[a], Annabel Cho[a,b], Ilknur Ay[a], Giorgio Bonmassar[a*]

[a] *Athinoula A. Martinos Center for Biomedical Imaging, Massachusetts General Hospital, Harvard Medical School, Charlestown, 02129, MA, United States*
[b] *Harvard University, Cambridge, MA, United States*

**Corresponding author: Giorgio Bonmassar, Ph.D., A. Martinos Center for Biomedical Imaging, MA, United States, Giorgio.Bonmassar@mgh.harvard.edu*

Contents

S1 Appendix. 2
S2 Appendix. 3
S3 Appendix. 4
S4 Appendix. 6
S5 Appendix. 9
S6 Appendix. 10
S7 Appendix. 14
S8 Appendix. 15
References 16

S1 Appendix.
The manuscript contains additional information on the segmentation process pipeline used to generate FIGURE 10.

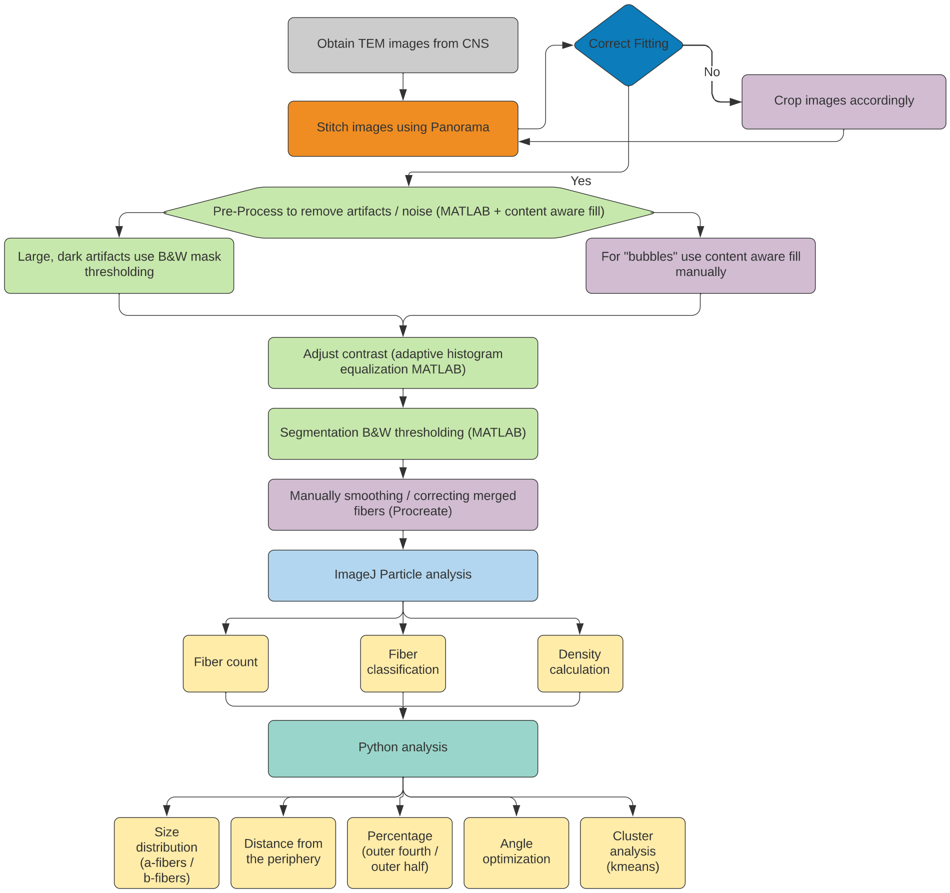

Supplemental Fig. S-1: *Segmentation flow chart describing the process of vagus nerve segmentation.*

S2 Appendix.

This section contains additional information on the RMS estimated |E|-field in the x-, y-, and z-directions on a nerve block 5µm under the µM-VNS coil with three spatial partial derivatives. The highest RMS |E|-field was 12.70 V/m, and the maximum gradient RMS |E|-field was 166,767 V/m^2, both located underneath the coil (Supplemental Fig. 2).

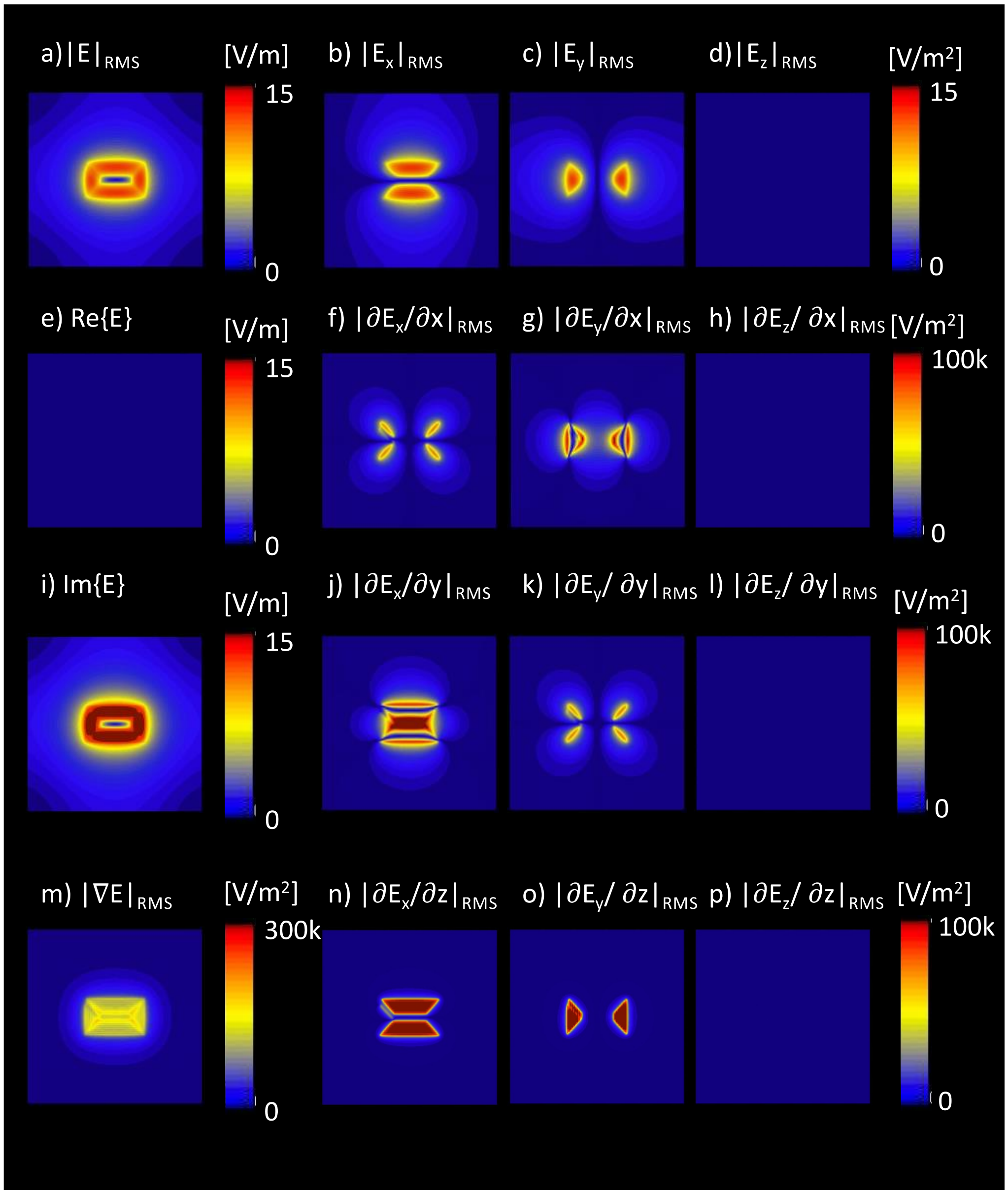

Supplemental Fig. S-2: *Top view of the |E|-field generation on the nerve surface with a) $|E|_{RMS}$, b) $|E_x|_{RMS}$, c) $|E_y|_{RMS}$, d) $|E_z|_{RMS}$, e) the strength of the real part of the E-field, and i) the strength of the imaginary part of the E-field; Top view of the spatial gradient of the $|E_x|_{RMS}$-field with f) x-, g) y-, and h) z- direction; the spatial gradient of the $|E_y|_{RMS}$-field with j) x-, k) y-, and l) z-direction, and the spatial gradient of the $|E_z|_{RMS}$-field with n) x-,o) y-, and p) z- direction; m) gradient of the $|E|_{RMS}$.*

S3 Appendix.
This section contains additional information on the experimental set-up and results of the sciatic nerve stimulation. The sciatic nerve stimulation experiments were conducted to check the efficacy of the magnetic nerve stimulation. The same experimental parameters were used for the micro-magnetic VNS (µM-VNS) and electrical VNS (eVNS) experiments (see the main manuscript). Adult male Wistar rats (350-420 g; Charles River Laboratories, Wilmington, MA) were anesthetized using isoflurane. The sciatic nerve was isolated, and a µM-coil was placed around the sciatic nerve in the hindlimb was immersed in a physiological solution. Electromyography (EMG) recordings were performed using needle electrodes (ADInstruments, USA) implanted into the gastrocnemius muscle in the left hindlimb of the rats in response to the ipsilateral sciatic nerve stimulation during electrical and magnetic stimulation. Data were acquired using BioAmp (ADInstruments, USA) connected to a PowerLab 8/35 (ADInstruments, Colorado Springs, CO, USA). LabChart software was used to analyze the data (acquisition rate 2000 samples per second using a mains filter).

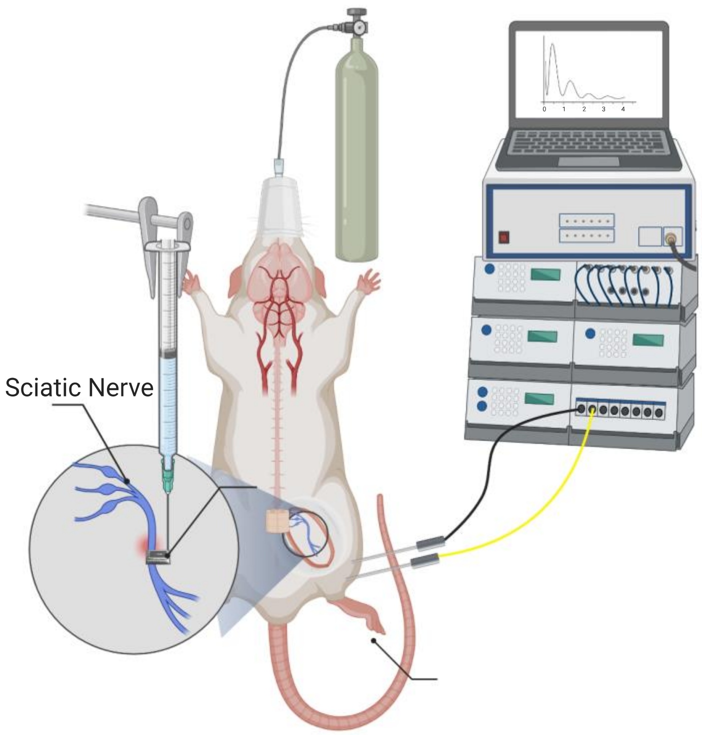

Supplemental Fig. S-3: *Experimental set-up used for the sciatic nerve stimulation. After anesthesia, the sciatic nerve was isolated and a µM-VNS coil was placed around the sciatic nerve. EMG was recorded from the gastrocnemius muscle (Figure created with http://biorender.com).*

In the electrical and magnetic stimulation cases, the EMG traces showed clear responses during the stimulation period. Although stimulus duration for eVNS and µM-VNS were similar (i.e., Δt=11ms for the eVNS, and 9 ms for the µM-VNS), M-waves were different for the two cases could be due to the different fiber recruitment levels.

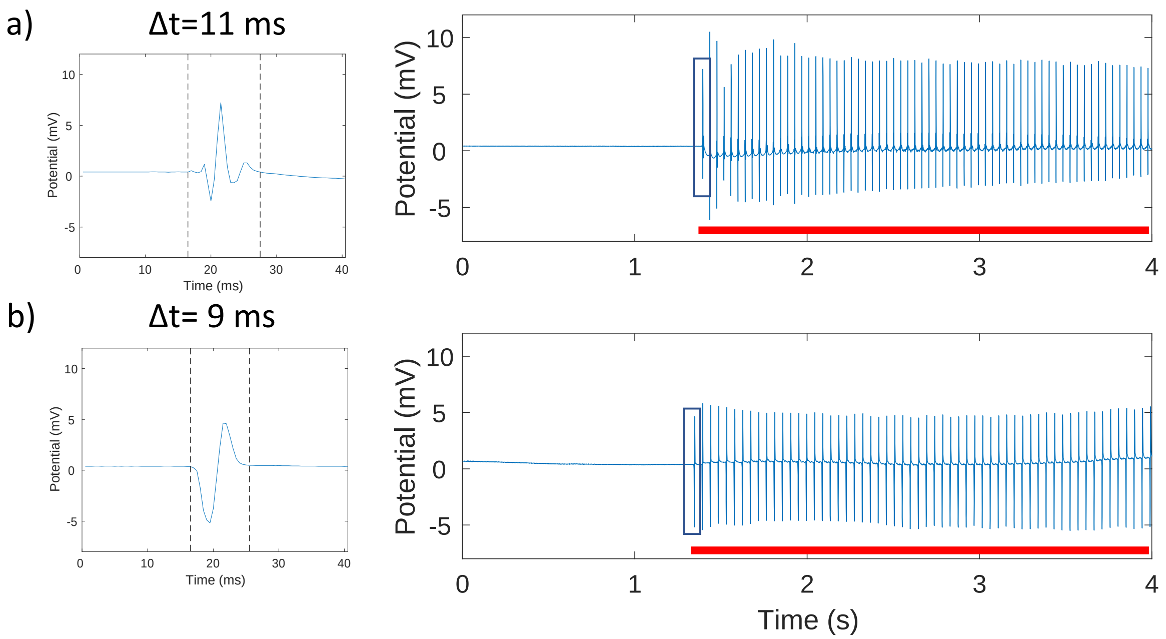

Supplemental Fig. S-4: *Sciatic nerve stimulation EMG measurements. The red line indicates the stimulation duration, and the blue box shows a single pulse; a) EMG response during electrical stimulation (0.5mA, pulse width: 0.5ms, 20Hz) and magnified view; b) EMG response during magnetic stimulation (V_{p-p}= 29.2 ±3.1 V, five exponential pulses train at 20Hz).*

S4 Appendix.

This paragraph contains additional information on the fusing of the planar spiral coil (PN LQP15MN 33nH SMD inductor, Murata Manufacturing Co., Japan) and the benchtop measurement results of the voltage, current, and time-varying magnetic field (dB/dt) using the short exponential pulse (pulse width= 10μs).

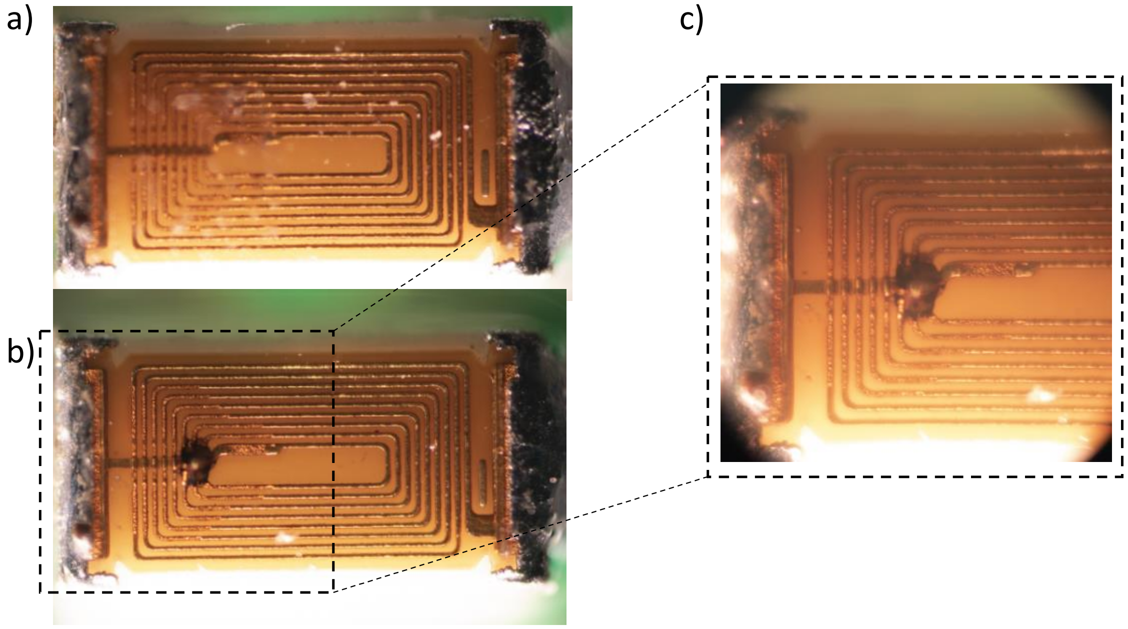

Supplemental Fig. S-5: *Fusing test results; a) Picture of the planar spiral coil before fusing test; b) after fusing test; c) zoomed view of the fused area near the wire that connects the soldering pad to the center of the planar spiral coil.*

The maximum input voltage and fusing current of the µM-VNS coil (PN LQP15MN 33nH SMD inductor, Murata Manufacturing Co., Japan) are shown in Supplemental Fig. S-6. The fusing test was done using a single exponential pulse at 20 Hz (i.e., 20 pulses per second) with various decaying pulse widths between 10 and 400 µs using the two signal generators from Tektronix (AFG1062, Tektronix, Beaverton, OR). The voltage was measured using a mixed-domain oscilloscope (MDO3024, Tektronix, Beaverton, OR). The time-varying magnetic field (i.e., dB/dt) was measured at a 1 mm distance from the coil using a magnetic near-field probe (PN PR262, B&K Precision, Yorba Linda, CA, USA) connected to an oscilloscope. The input current was estimated using a hall effect (Huber et al., 2015) sensor (ACS772 200B series, Allegro Microsystems, Inc.).

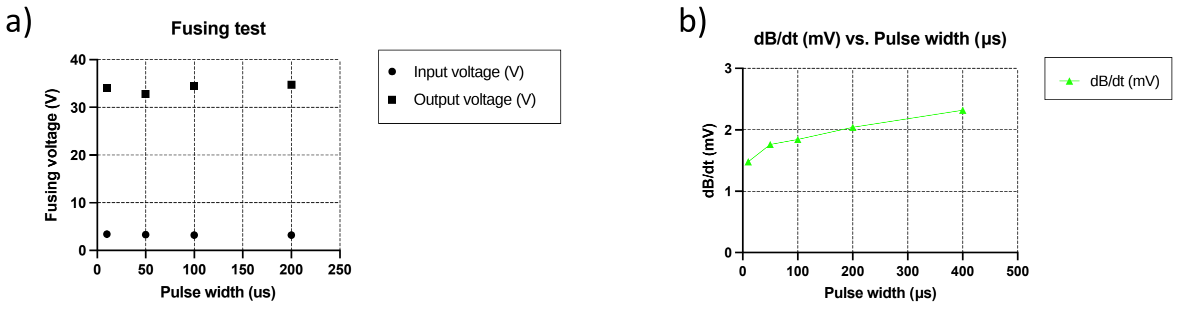

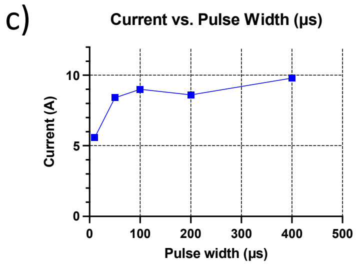

d)

Parameter	Threshold
Fusing voltage	> 35 V
Fusing current	> 10 A

Supplemental Fig. S-6: Fusing test results; The µM-VNS coil was tested with a single exponential pulse with different pulse widths until trace fuses; a) shows the input voltage from the function generator and measured output voltage from the class-D amplifier, b) shows the measured time-varying magnetic field across the series of pulse widths, and c) shows the measured current across series of pulse widths, and d) shows the fusing voltage and current.

The exponential pulse response was measured using a single exponential pulse at 20 Hz (i.e., 20 pulses per second) with pulse widths of 10 µs using the two signal generators from Tektronix (AFG1062, Tektronix, Beaverton, OR, USA). The voltage was measured using a mixed-domain oscilloscope (MDO3024, Tektronix, Beaverton, OR, USA). The time-varying magnetic field (i.e., dB/dt) was measured at a 1 mm distance from the coil using a magnetic near-field probe (PN PR262, BK Precision, Yorba Linda, CA, USA) connected to an oscilloscope. The input currents were estimated using a hall effect (Huber et al., 2015) sensor (ACS772 200B series, Allegro Microsystems, Inc.).

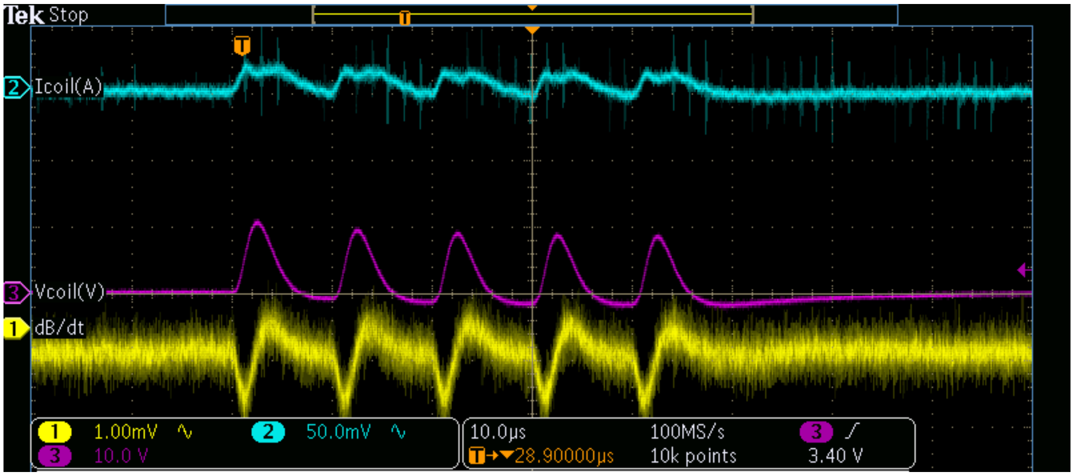

Supplemental Fig. S-7: *Benchtop dB/dt, current, and voltage test results of the exponential pulse. Channel 1 (Yellow) shows the dB/dt probe (measuring distance = appx. 1 mm from the coil surface); Channel 2 (blue) shows the results of in-house current measurement; Channel 3 (violet) shows the measured voltage.*

S5 Appendix.

This section contains additional information on the experimental data of eVNS and µM-VNS from Wistar rats. The normality of the data was tested using the *Shapiro-Wilk test.* Comparisons within groups were performed using a 2-way analysis of variance (ANOVA) followed by Tukey's multiple comparison test when needed for data that showed normal distribution. *Multiple Wilcoxon test* was used when the data were not normally distributed. $P < 0.05$ was considered significant. Data were expressed as mean ± standard error of the mean.

		eVNS	µM-VNS
ABP (mmHg) F(1,7) = [37.85]	Before stimulation	112.29 ± 9.46	104.46 ± 16.76
	During stimulation	76.68 ± 19.77	103.95 ± 15.67
	Percent difference	-32.39 ± 12.84 %*	-0.32 ± 2.42% (n.s.)
RR (breaths/min) F(1,7) = [60.82]	Before stimulation	34.60 ± 12.46	27.22 ± 9.10
	During stimulation	7.29 ± 4.61	13.86 ± 7.66
	Percent difference	-77.04 ± 11.03%*	-50.11 ± 16.19%*
HR (heartbeats/min)	Before stimulation	314.22 ± 60.63	286.70 ± 29.45
	During stimulation	286.70 ± 29.45	285.93 ± 30.49
	Percent difference	-55.29 ± 18.79 %*	-0.30 ± 0.46% (n.s.)
	q value (Wilcoxon test)	0.015781	0.189375

Supplemental Table S-1: *Physiological responses and statistical responses to eVNS and µM-VNS. Respiration data was smoothed with a triangular (Bartlett) window (0.8s width) and used for calculating the respiration rate with a 'respiratory belt' preset with a minimum peak height standard deviation of 0.9 in the cyclic measurement tool in LabChart. Heart rate was calculated with 'ECG – rat' preset with a minimum peak height standard deviation of 1.5 in the cyclic measurement tool in LabChart. * $p < 0.05$ before vs. during stimulation.*

S6 Appendix.

This section contains additional information of the codes used for the figure generation.

```
# import libraries
import numpy as np
import matplotlib
import matplotlib.pyplot as plt
import scipy.stats
import pandas as pd
from mpl_toolkits.mplot3d import Axes3D
%matplotlib inline

# change the default font size of plots to 25
matplotlib.rcParams.update({'font.size': 50})

# Importing RAT A data
diameter_median=2.98
diameter_ratA = np.loadtxt('diameter_ratA')
b_fiberA = np.array(diameter_ratA) < diameter_median
a_fiberA = np.array(diameter_ratA) > diameter_median

# Importing RAT B data
diameter = np.array(pd.read_csv('Results1.csv')["Diameter"])
pd.read_csv('Results1.csv')
b_fiber = np.array(diameter) < diameter_median
a_fiber = np.array(diameter) > diameter_median

# Importing 1-20-6 RAT C
diameter_ratC = np.loadtxt('diameter_ratC')
b_fiberC = np.array(diameter_ratC) < diameter_median
a_fiberC = np.array(diameter_ratC) > diameter_median

# 3d figure start
fig = plt.figure(figsize=(20,20))
ax = fig.add_subplot(111, projection='3d')

# parameter changes
```

```
afib_bin = 15
bfib_bin = 4

# Rat A, Large fibers
ys = diameter_ratA[a_fiberA]
hist, bins = np.histogram(ys, bins=afib_bin)
xs = (bins[:-1] + bins[1:])/2
ax.bar(xs, hist, zs=5, zdir='y', color='b', ec='b', alpha=0.8)

# Rat A, Small fibers
ys = diameter_ratA[b_fiberA]
hist, bins = np.histogram(ys, bins=bfib_bin)
xs = (bins[:-1] + bins[1:])/2
ax.bar(xs, hist, zs=5, zdir='y', color='r', ec='r', alpha=0.8)

# Rat B, Large fibers
ys = diameter[a_fiber]
hist, bins = np.histogram(ys, bins=20)
xs = (bins[:-1] + bins[1:])/2
ax.bar(xs, hist, zs=10, zdir='y', color='b', ec='b', alpha=0.8)

# Rat B, Small fibers
ys = diameter[b_fiber]
hist, bins = np.histogram(ys, bins=4)
xs = (bins[:-1] + bins[1:])/2
ax.bar(xs, hist, zs=10, zdir='y', color='r', ec='r', alpha=0.8)

# Rat C, Large fibers
ys = diameter_ratC[a_fiberC]
hist, bins = np.histogram(ys, bins=27)
xs = (bins[:-1] + bins[1:])/2
ax.bar(xs, hist, zs=15, zdir='y', color='b', ec='b', alpha=0.8)

# Rat C, Small fibers
ys = diameter_ratC[b_fiberC]
hist, bins = np.histogram(ys, bins=4)
xs = (bins[:-1] + bins[1:])/2
ax.bar(xs, hist, zs=15, zdir='y', color='r', ec='r', alpha=0.8)
```

```
ax.set_xlabel('\n\nDiameter (Âµm)')
ax.set_zlabel('\n\nCount')
ax.set_xlim([0,25])

#removing y axis
ax.set_yticklabels( () )

# Labels
ax.text(0, 5, 780, "Rat A", color='k')
ax.text(0, 10, 550, "Rat B", color='k')
ax.text(0, 15, 630, "Rat C", color='k')

# manually set legend
import matplotlib.patches as mpatches
red_patch = mpatches.Patch(color='red', label='Small Fibers')
blue_patch = mpatches.Patch(color='blue', label='Large Fibers')
plt.legend(handles=[blue_patch, red_patch], loc=1, shadow=True, edgecolor='k')

plt.savefig('3d_hist.pdf', bbox_inches='tight')
plt.show()
```

Supplemental Code 1: *Python code used for figures generation (FIGURE 10B).*

```
% K-mean cluster
rng default; % For reproducibility
X=[Fiber_location(:,:); Weight;];
figure;plot(X(:,1),X(:,2),'.');axis image;
nC=11; fts=24;
opts = statset('Display','final');
[idx,C] = kmeans(X,nC,'Distance','cityblock', 'Replicates',5,'Options',opts);
m_sz=16; %marker size
figure;
plot(X(idx==1,1),X(idx==1,2),'r-','MarkerSize',m_sz); hold on
plot(X(idx==2,1),X(idx==2,2),'b-','MarkerSize',m_sz); hold on
plot(X(idx==3,1),X(idx==3,2),'g-','MarkerSize',m_sz); hold on
plot(X(idx==4,1),X(idx==4,2),'k-','MarkerSize',m_sz); hold on
plot(X(idx==5,1),X(idx==5,2),'c-','MarkerSize',m_sz); hold on
plot(X(idx==6,1),X(idx==6,2),'m-','MarkerSize',m_sz); hold on
plot(X(idx==7,1),X(idx==7,2),'-','Color',[0.93 0.69 0.13],'MarkerSize',m_sz);hold on
plot(X(idx==8,1),X(idx==8,2),'-','Color',[0.85 0.33 0.10],'MarkerSize',m_sz);hold on
plot(X(idx==9,1),X(idx==9,2),'-','Color',[0.49 0.18 0.56],'MarkerSize',m_sz);hold on
plot(X(idx==10,1),X(idx==10,2),'-','Color',[0.47 0.67 0.19],'MarkerSize',m_sz);hold on
plot(X(idx==11,1),X(idx==11,2),'-','Color',[0.47 0.19 0.67 ],'MarkerSize',m_sz);
plot(C(:,1),C(:,2),'kx', 'MarkerSize',m_sz+4,'LineWidth',3)
legend('Cluster 1','Cluster 2','Cluster 3','Cluster 4','Cluster 5','Cluster 6','Cluster 7','Cluster
8','Cluster 9','Cluster 10','Cluster 11','Centroids', 'Location','SE');
set(gca,'Xticklabel',get(gca,'Xtick'),'Yticklabel',get(gca,'Ytick'),'fontsize',fts);
set(gca,'YDir','reverse'); hold off
```

Supplemental Code 2: *MATLAB code used for k-mean clustering (FIGURE 11).*

S7 Appendix.
This paragraph contains additional information on the k-means clustered on large fiber groups of three rats. (see FIGURE 11 in the manuscript for the non-overlaid result).

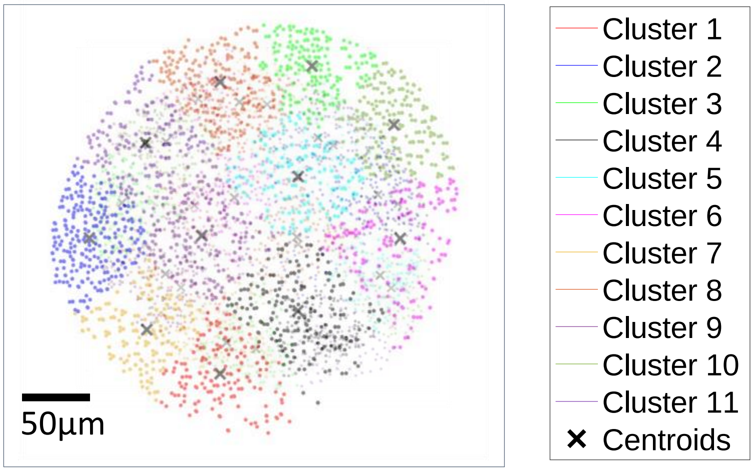

Supplemental Fig. S-8: *superimposed clustering results of k-means clustered on large fiber group of three rats.*

S8 Appendix.
This paragraph contains additional information on the µM-VNS coil position in the animal experiment.

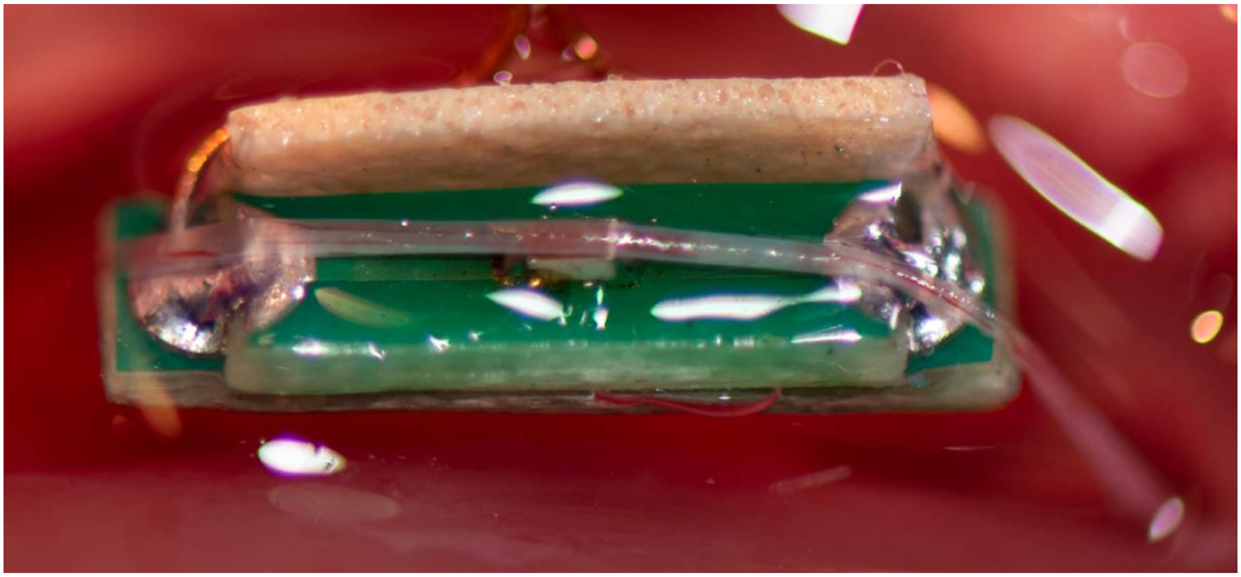

Supplemental Fig. S-9: *µM-VNS coil positioned underneath the vagus nerve in the animal experiment.*

References

Huber, S., Leten, W., Ackermann, M., Schott, C., and Paul, O. (2015). A Fully Integrated Analog Compensation for the Piezo-Hall Effect in a CMOS Single-Chip Hall Sensor Microsystem. *IEEE Sens. J.* 15, 2924–2933. doi:10.1109/JSEN.2014.2385879.
